# Microneedle-based injection of Fungizone/Amphotericin B: an effective treatment for American cutaneous leishmaniasis in mice

**DOI:** 10.1080/10717544.2026.2665882

**Published:** 2026-05-04

**Authors:** Ryan H. Huston, Blake Cox, Chaitenya Verma, Thalia Pacheco-Fernandez, Greta Volpedo, M. Junaid Dar, Yulian Mercado, Abigail R. Wharton, Max Gessner, Nandu Purayil, Ivana Arsovska, Leah Watkins, Junqi Lu, Jennifer Y. Zhang, Andrew Sachan, Roger J. Narayan, Abhay R. Satoskar

**Affiliations:** a Department of Microbiology, The Ohio State University, Columbus, OH, USA; b Department of Pathology, Wexner Medical Center, The Ohio State University, Columbus, OH, USA; c Department of Dermatology, Duke University, Durham, NC, USA; d Department of Pathology, Duke University, Durham, NC, USA; e Joint Department of Biomedical Engineering, University of North Carolina at Chapel Hill and North Carolina State University, Raleigh, NC, USA

**Keywords:** Microneedle, transdermal drug delivery, Amphotericin B, cutaneous leishmaniasis, *Leishmania*

## Abstract

Amphotericin B (AmB) is a potent, accessible, FDA-approved drug against CL with damaging side effects. While newer lipid formulations have less toxicity, limitations include affordability and cold-chain requirements. CL affects mostly impoverished communities, which complicates travel to urban hospitals for treatment. Therefore, an off-clinic drug delivery system is desirable. Microneedles can deliver AmB directly to localized CL lesions, which we hypothesized would limit parasite growth without systemic toxicity. Thus, a three-by-three array of 1 mm tall stainless steel hollow microneedles was evaluated in this study. To test their efficacy, we utilized *Leishmania mexicana-*infected mice to model American CL. Following 20- and 10-consecutive day microneedle treatments (*n* = 5/group), the 20-day trials more strongly limited lesion growth by up to 2.8 mm versus a maximum 1.1 mm difference between the drug and placebo groups in the 10-day trial (*p* = 0.008, *p* = 0.075, respectively). Also, treatments were associated with reduced parasitic burden weeks after treatment cessation, as assessed by limiting dilution (*p* = 0.057 for 10-day, *p* = 0.075 for 20-day; one-tailed Mann‒Whitney *U* test). No elevation of blood serum creatinine or blood urea nitrogen levels was observed, supporting the hypothesis of lessened kidney toxicity. Additionally, flow cytometry explorations showed differences in IL-17 and IFN-γ positivity, and histological changes were observed in the upper dermis. These promising results represent the first test of hollow microneedles for CL treatment and pave the way for further trials. The adoption of microneedles could reduce CL burden in affected communities because of their ease of use, efficacy, and safety with a pre-approved drug.

## Graphical abstract

**Figure:**
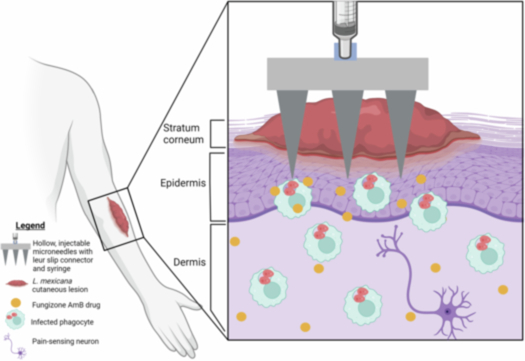

## 1. Introduction

Despite global efforts, cutaneous leishmaniasis (CL) causes more than one million new infections annually, in addition to the 4–10 million current untreated cases; the burden for CL in terms of disability-adjusted life years has been on the rise since 1990 (Bailey et al. 2017; Vos et al. 2020; WHO 2022b). CL is caused by infection with parasites of the genus *Leishmania*, and endemic regions include 90 countries, mostly in the tropics (WHO 2022b). CL in Mexico and Central America is most frequently caused by *Leishmania mexicana*, which was recently declared endemic to the USA (Aronson et al. 2016; Monroy-Ostria and Sanchez-Tejeda 2017; McIlwee et al. 2018; WHO 2022a). Currently, there is no vaccine for human leishmaniasis; thus, the only strategies to limit the spread involve treating patients and limiting contact with vector species (Volpedo et al. 2021). *L. mexicana* infection often manifests as a single skin ulceration at the site of the sandfly vector bite, in which case it is considered localized CL (LCL); other presentations of CL also exist (McGwire and Satoskar 2014; Handler et al. 2015; Volpedo et al. 2021). Although CL is rarely fatal, the disfiguration can often result in social isolation due to various stigmas, which can create elevated anxiety, depression, and suicidal thoughts (Okwor and Uzonna 2016; Bennis et al. 2018; Boukthir et al. 2020; Aghakhani et al. 2023). CL is more prevalent in underdeveloped rural areas with inadequate healthcare facilities, which means that patients often wait longer and must travel farther to seek treatment (Okwor and Uzonna 2016; Weiss et al. 2020). Travel distance also leads to disruption of patients' lives during week-long treatment, which leads to lower compliance and additional financial burden (Hotez et al. 2004). Hence, improved affordable and accessible treatments are still needed for this neglected tropical disease to improve patient compliance and reduce transmission of cutaneous leishmaniasis.

Treatments exist for CL, but all of them have drawbacks. One of the most prescribed drugs, Amphotericin B (AmB), is often formulated with the solvent deoxycholate, which is commercially known as Fungizone (Fungizone AmB in this paper) (Ponte-Sucre et al. 2017; Wijnant et al. 2018). AmB is a polyene that targets ergosterol in leishmanial membranes and can cure CL (Croft et al. 2006); as Fungizone, it also often causes severe side effects such as fever, seizures, organ failure, and even death (Bates et al. 2001; Hamill 2013; Ponte-Sucre et al. 2017). In some cases, patients who experience toxicity from Fungizone AmB will cease treatment before parasite clearance, which could lead to AmB-resistant parasite emergence, as has been observed for *L. mexicana in vitro* (Al-Mohammed et al. 2005; Ponte-Sucre et al. 2017; Mwenechanya et al. 2017; Pountain et al. 2019; Pountain and Barrett 2019; Alpizar-Sosa et al. 2022). Other liposomal Amphotericin B (L-AmB) formulas, such as Amphocil and AmBisome, have also been developed and tested for leishmaniasis to reduce toxic side effects (Yardley and Croft 2000; Hamill 2013; Wijnant et al. 2018). Both Fungizone and L-AmB have low bioavailability orally and therefore must be injected intravenously (IV), requiring hospitalization for CL treatment (Hamill 2013; Rodríguez Galvis et al. 2020; Wasan et al. 2022). Specifically, Fungizone treatment requires up to 30 consecutive days of treatment, whereas L-AmB for CL is administered for up to 21 days on alternate days (Rodríguez Galvis et al. 2020). However, L-AmB is more expensive and requires cold-chain storage, which makes it difficult for poorer, rural populations to access, especially where power is not consistently available (Ponte-Sucre et al. 2017). Thus, owing to its affordability and stability, Fungizone AmB is still widely used to treat CL, and safety problems (e.g. kidney and liver toxicity) remain risks.

Drug administration by microneedles instead of by IV could address major issues for CL treatment, similar to current intralesional injection methods (Aronson and Joya 2019). Microneedles are miniature versions of the needles used in IV injections, and are typically grouped in arrays. The depth of microneedle penetration ranges from 0.15 to 3 mm; these devices go through the stratum corneum and epidermis but often avoid deeper dermal pain receptors (Sabri et al. 2019; Shukla et al. 2022). Therefore, microneedles have been shown to be a simple and relatively painless technique for potential use in at-home administration, as reported in human trials (Gupta et al. 2011; Arya et al. 2017). Transdermal microneedle drug delivery has even been shown to enable systemic drug delivery through diffusion to capillaries within the skin (Prausnitz and Langer 2008; Wermeling et al. 2008; Hultström et al. 2014; Chen et al. 2018). Microneedles can use multiple strategies to bypass the major barrier of the stratum corneum and endogenous skin oils, which otherwise limits topical drug effectiveness and penetration depth (Shukla et al. 2022). For example, solid microneedles can make microscopic incisions where topical medication can enter the skin, the drug can be coated on the needles, biodegradable microneedles can release the drug as they dissolve, or hollow microneedles can directly inject medication into the skin. These delivery strategies could be analogous to intradermal injections or even intravenous injections when given at sufficient doses. Each class of microneedle has been investigated across numerous applications, diseases, and with thousands of patients within clinical trials, as reviewed previously (Ingrole et al. 2021), with no reported significant risk of infection or other major complications so far (Dul et al. 2023).

Thus far, investigations into the efficacy of microneedle-based treatments for CL have been largely positive. *In vitro* and *ex vivo* studies have indicated that solid microneedles enhance superficial drug penetration for a novel AmB, but there is no benefit to deeper dermal layers (Fernández-García et al. 2020); meanwhile, dissolvable microneedles of various formulations have been demonstrated to have potential utility against *Leishmania* *major* (Zare et al. 2021; Dastan et al. 2025) and *in vitro* against *Leishmania* *donovani* (Wang et al. 2025). However, only one prior study tested microneedles for *in vivo* treatment of LCL of *L. mexicana*. Nguyen et al. found that solid microneedles combined with a non-commercial AmB formulation outperformed topical administration alone and performed comparably to IV L-AmB treatment for *L. mexicana* infection but saw no benefit for treating *L. major* infection in the BALB/c mouse model (Nguyen et al. 2019). For practical translation, however, the evaluation of a commercially available drug such as Fungizone would be desirable. Thus, in the present study, we evaluated a novel hollow microneedle device to inject commercially available Fungizone AmB to treat *L. mexicana* infection in BALB/c mice, with the hypothesis that lesion size and parasitic burden are reduced. Hollow microneedles have not been previously tested for use in CL treatment but have been tested for vaccine delivery (van der Maaden et al. 2014), cancer therapeutics (Abd-El-Azim et al. 2022), bacteriophages (Ryan et al. 2012), and other agents. In LCL, we demonstrated that hollow microneedles penetrate the stratum corneum and outermost layers of the epidermis, allowing for the injection of the drug directly into the CL lesion site, reducing disease symptoms, reducing parasite burden, and limiting systemic toxicity.

## 2. Materials & methods

### 2.1. Microneedle array

**Figure 1.**
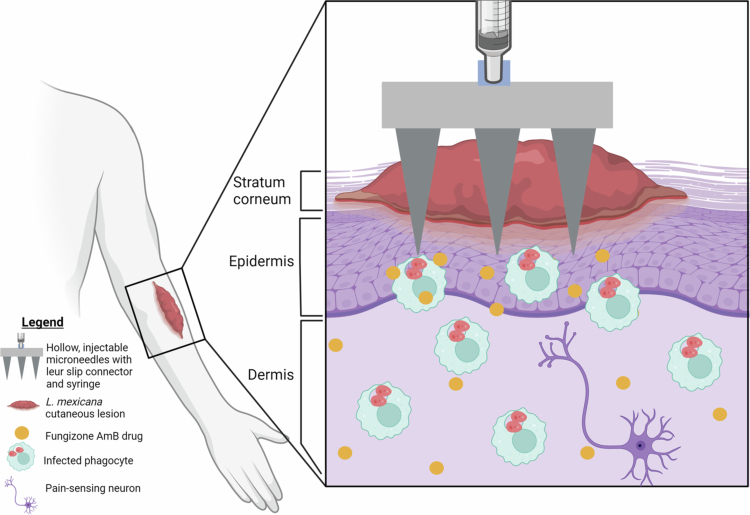
Hollow microneedle overview of structure and injection of Fungizone AmB into *L. mexicana* lesion. The microneedle device is constructed with a Luer slip connector that attaches to a conventional compatible syringe and allows for injection of the drug through hollow needles directly into the epidermis, bypassing the stratum corneum. Elements in the illustration, including cell types, may not be to scale.

The hollow microneedle array consists of nine microneedles composed of AISI 304 stainless steel and a polypropylene body in a three-by-three orientation with an attached female luer slip connector; this connector can be attached to a 1 mL conventional syringe (Micropoint Technologies Pte Ltd., Pioneer Junction, Singapore), as diagrammed (Figure 1). The microneedle height is 1 mm, the inner diameter of the hollow bore is 150 µm, and the outer diameter of the hollow bore is 250 µm; the pitch (the distance between two microneedles in the nine-microneedle array) is 3.5 mm. The height of the microneedle is sufficient to enter the stratum corneum and deeper layers of the epidermis (Monteiro-Riviere et al. 1990) for the transdermal delivery of drugs (Papich and Narayan 2022). After use, microneedles are disposed of as biohazardous sharps, similar to conventional syringe needles.

### 2.2. Imaging of the microneedle array

Scanning electron microscopy (SEM) measurements to understand the microstructure of the microneedles in the microneedle array were undertaken using a FEI Helios 600 NanoLab system, which includes a field emission scanning electron microscope (FE-SEM) and a focused ion beam (FIB) instrument (Thermo Fisher, Waltham, MA, USA). The scanning electron microscope was operated at an acceleration voltage of 5 kV, a beam current of 0.69 nA, and a working distance of approximately 5 mm. The sample was attached using double-sided carbon tape to an aluminum sample holder. A 5 nm gold‒palladium alloy conductive coating was deposited on the surface of the sample prior to imaging.

### 2.3. Optical imaging of interactions of the microneedle array with porcine skin

Porcine skin without subcutaneous fat was obtained from a commercial source (Vincent's Meat Market, Bronx, NY, USA). After thawing, the skin was rinsed with phosphate-buffered saline (PBS) and dried using Kimwipe (Kimberly Clark Professional, Roswell, GA, USA). The skin was then placed in a Petri dish with the epidermis side facing up. A 1 mL syringe was loaded with 500 μL of 1% methyl blue; the microneedle array was attached to the syringe. With the microneedle array pointed up, the plunger was slid carefully to remove any air inside the syringe. The skin was punctured with the microneedle array to inject the dye. The injection process was repeated several times on the skin. After 30 seconds, the skin was flushed with PBS to remove any excess dye and dried again with a Kimwipe. Around the needle points, a 1 cm-by-1 cm tissue block was cut out, submerged in the optimal cutting temperature compound, frozen on dry ice, and kept at −80 °C until sectioning. A cryostat microtome was used to prepare a series of 7 μm-thick skin sections. The sections with microneedle tracks were then imaged using BX41 microscopic imaging equipment with the brightfield setting (Olympus, Center Valley, PA, USA).

### 2.4. Optical coherence tomography imaging of interactions of the microneedle array with porcine skin

Optical coherence tomography (OCT) was used to examine the penetration of commercially obtained porcine skin by the microneedle array with micrometer-level spatial resolution (Shrestha and Stoeber 2018; Plamadeala et al. 2019). This was done with three different Thorlabs OCT systems – OCT-LK3 objectives were utilized with the VEG210 and TEL221 systems; an OCT-LK3-BB objective was utilized with the GAN611 system. All the objectives provided a 10 × 10 mm field of view. These systems provide an image depth of 2.9 mm in air. It should be noted that the imaging depth is reduced in materials with a high degree of scattering, such as the skin. The microneedle array was pressed against the porcine skin. The microneedles were injected at an angle, such that the microneedles entered ~400 µm into the tissue. A measurement was recorded with microneedles inserted into the porcine skin. Another measurement was recorded from bare porcine skin after removing the microneedles.

### 2.5. Parasite cultures

*L. mexicana* (MNYC/B2/62/m379) parasites were maintained via subcutaneous injection in the shaved back rumps of female 129S6/SvEvTac mice as needed, aged 7 weeks or older, which were purchased from Taconic Biosciences, Inc. Once lesion development became advanced or when promastigotes were needed to begin the experiment, the stock mice were euthanized, and amastigotes were obtained from single-cell suspensions, which were acquired from the draining lymph nodes. The parasites were then differentiated into promastigotes via *in vitro* culture in M199 medium supplemented with 10% fetal bovine serum (FBS), 1% penicillin/streptomycin, and 10 mM HEPES at 26 °C and passaged 3‒6 times to generate stationary phase promastigotes before use in murine experimental infections.

### 2.6. Mouse colony

Mice were chosen for our *in vivo* model because they are accessible and established models for cutaneous leishmaniasis. Female BALB/c mice were obtained from a commercial source (Envigo (Harlan Laboratories), Indianapolis, IN, USA). The BALB/c model was chosen because of the formation of non-healing LCL lesions during *L. mexicana* infection; thus, the positive effects are only due to treatment (Rosas et al. 2005). All the mice were housed at the Ohio State University animal facility, following the approved animal protocols and University Laboratory Animal Resources (ULAR) regulations (Protocol 2022A00000030, which contains approved protocols for multiple studies). Each experiment was initiated with 10 age-matched 5–8-week-old female mice or older; 5 mice were examined per group to compare drug and placebo treatments. Two age-matched naïve mice were also used for histology controls in the 10- and 20-day trials. This sample size was chosen in line with previous studies to observe biological effects while limiting the number of animals (Nguyen et al. 2019). Over our 3 trials, a total of 34 mice were used. The typical weight of female BALB/c mice over the ages used at the beginning of these trials was 25 g. The 3 independent trials were conducted between 8/6/2021 and 5/21/2025.

### 2.7. Mouse infection, monitoring

Aged-matched BALB/c mice were infected via an intradermal injection of 1 × 10^6^ *L. mexicana* P3-6 promastigotes in 100 µL of saline, which was injected into the shaved back rump to create the skin lesions for treatment. The infected mice were monitored until the lesions in all the mice from both groups exceeded 2 mm in diameter, which was our only inclusion criterion. There were no exclusion criteria predetermined, and no mice were excluded. Once this criterion was met for all the mice, all the lesions were measured, and the mice were manually randomized such that each group had a roughly equal starting average lesion size. After this time, the lesions were measured weekly as described below. Three independent trials were run, one for the 10-day treatment and two for the 20-day microneedle treatment.

The back rump CL lesion size was monitored using a Thickness Gauge (Standard Type) Teclock (scale interval 0.01 mm, range 0–20 mm). To ensure consistency, the mice were measured by a single trained user in each trial following a standard procedure. Both the vertical diameter and horizontal diameter were measured, similar to prior studies (Dar et al. 2020), and the ‘size’ was the average of these two measurements. In a few cases, two lesions occurred on the rump of the same mouse; in these cases, the lesions were measured separately, and the size of each lesion was added together to make them comparable with those of the other mice.

### 2.8. Mouse blood draws and treatments

Once the lesions were of a suitable size as described above, blood was obtained in non-heparinized tubes (Lafuse et al. 2013) from all the mice on the day before the start and after the end of treatment via a shallow tail vein cut, a restraining device, and no anesthesia (Virginia Tech, 2017). The murine blood was stored at 4 °C overnight and then spun to pellet blood cells, after which the serum was isolated as previously described (Volpedo et al. 2022). The serum was then isolated and stored long-term at –20 °C. During the treatment periods, hollow microneedle devices were employed daily (10 or 20 days) to inject either a commercial Fungizone Amphotericin B formulation (ThermoFisher cat# 15290018) or a placebo control. For Fungizone AmB, 1 mg/kg/day (100 µL) was delivered as measured by the 1 mL syringe volume markings. This dose is comparable to those used in prior studies with alternate injection methods (Khan and Owais 2006; Tavares et al. 2019). For the placebo control solution, an equivalent volume was given at the same concentration of the solvent sodium deoxycholate (bioWORLD cat# 40430018-2) mixed with dH_2_O, as is used in Fungizone. These solutions were loaded into the microneedles via a 1 mL syringe with a male luer slip connector, which attaches directly to the hollow microneedle device. The microneedles and syringe were loaded with the solution and combined carefully without air bubbles. Then, the solution was pressed out of the syringe through the microneedles so that small droplets could be seen on the needle tips to ensure the quality of the needles and that there was no clogging. New microneedle devices were used for each group on each day, which amounts to using the same needle 5 times before disposal. Finally, the mice were held in place by their tail while the microneedles were pressed firmly over the center of their lesion for drug or placebo injection into the skin.

Cages were coded to obscure group identities, but it was not possible to fully blind those measuring lesions and giving the drug treatments owing to the distinct color of the drug; this could be one limitation and source of bias. Placebo mice were treated first, followed by drug mice to minimize variability in treatment timing. Cage locations were adjacent to one another, and efforts were made to keep all the environmental factors as consistent as possible between groups.

### 2.9. Mouse handling

Once the groups no longer showed trends of statistical differences in lesion size, the mice were euthanized via CO_2_ asphyxiation, which was followed by cervical dislocation to confirm death. This endpoint always occurred before lesions reached established early removal criteria (progression to open ulcerating lesions with discharge or signs of secondary infection). Following our criteria for trial start and end, the endpoints varied slightly for each trial as follows: the 10-day treatment trial was concluded at 139 d.p.i. (41 days since the end of treatment), a 20-day treatment trial was concluded at 97 d.p.i. (26 days since the end of treatment), and the 20-day repetition trial was concluded at 99 d.p.i., 18 days post-treatment. At these endpoints, photos of the final lesions were documented. Subsequently, the cutaneous lesion, proximal lymph nodes (primarily subiliac), and spleen were collected. Half of the lesion tissue and all the spleen and draining lymph nodes were homogenized through a 70 µm cell strainer to create a single-cell suspension in 3 mL of RPMI supplemented with 10% heat-inactivated FBS and 1% penicillin/streptomycin. Spleens were passed through an additional step of isotonic red blood cell lysis, as previously described (Pérez-Santos and Talamás-Rohana 2001). The live cell density of these samples was then counted so that the cell number could be normalized in subsequent experiments, including parasitic burden, limiting dilution, and flow cytometry tests, as established previously (Volpedo et al. 2022).

### 2.10. Parasitic burden via limiting dilution

Parasitic burden was determined via the limiting dilution method as previously described (Ghotloo et al. 2015; Haghdoust et al. 2020). Single-cell suspensions were obtained in step 2.9 and resuspended in complete Schneider's Drosophila medium (Gibco, US) supplemented with 20% heat-inactivated FBS and 1% penicillin/streptomycin. Uniform cell concentrations were used within each trial. The cells were serially diluted 23 times (10-fold dilution step) across two 96-well plates and in duplicates. After 7 and 14 days of incubation at 26 °C, the plates were examined using an inverted microscope at a total magnification of 400x to observe promastigote parasite growth. The values that are reported in the graphs represent the highest log dilution, with the observed promastigotes at day 14 indicating recovery, differentiation, and *in vitro* growth of viable amastigote parasites into promastigotes.

### 2.11. RNA extraction and cDNA synthesis

The tissue samples were preserved in RNAlater (Invitrogen cat# AM7020) until use. Then, the tissue samples were washed with PBS and resuspended in 500 µL of TRIzol (Ambion cat# 15596-018) and homogenized. RNA extraction was performed according to the manufacturer's instructions. Sample concentrations were determined with a Nanodrop 1000 spectrophotometer (Thermo Scientific). cDNA synthesis was performed using the iScript cDNA synthesis kit (Bio-Rad cat# 1708891) following the manufacturer's instructions using 180.15 ng of input RNA in a 20 μL reaction.

### 2.12. Parasitic burden via RT-qPCR

PCR for the constitutive gene *Leishmania* α-tubulin (Fong et al. 1984) (F:5ʹ-GGC TTT ATG GTG TTC CAC GC-3ʹ, R:5ʹ-AAG CTT GGA CTT CTT GCC GT-3ʹ) and the murine housekeeping *β*-actin (F:5ʹ-TGG AAT CCT GTG GCA TCC ATG AAA-3ʹ, R:5ʹ-TAA AAC GCA GCT CAG TAA CAG TCC G-3ʹ) were performed using iQ™ SYBR® Green Supermix (Bio-Rad, cat# 1708882) per the manufacturer's instructions using 1 μL of cDNA. The amplification program used was 95 °C for 3 minutes, followed by 40 cycles of denaturation at 95 °C for 10 seconds and amplification at 55 °C for 30 seconds. Amplification and calculation of Ct values were carried out using the Bio-Rad C1000 Touch Thermocycler and CFX96 Real-Time System. Melt curve analysis was used as a quality control step – only reactions with a clearly dominant melting peak consistent with specific amplification were included. Ct values were then normalized within each mouse cDNA sample to the corresponding Ct values of murine *β*-actin, an established baseline gene. The primer efficiency was calculated via the LinRegPCR online toolkit (https://www.gear-genomics.com) (Untergasser et al. 2021), and the average primer efficiency (E-avg) was found to be 1.897. The efficiency-corrected fold change was then calculated per the MIQE 2.0 guidelines (Bustin et al. 2025). Therefore, E_avg_^−ΔΔCt^ = Efficiency-adjusted fold change of parasite α-tubulin expression relative to that in the placebo control group.

### 2.13. Histology

At the time of euthanasia, the skin lesion was excised from the rump of the mice. The lesions were round or oval shaped; the middle was cut down along a sagittal plane, and half of the lesion was fixed and stored in 4% paraformaldehyde. The tissue was then routinely processed on a Leica Histocore Peloris Tissue Processor, embedded in paraffin wax, sectioned in the sagittal plane so that the stratum corneum and deeper layers of skin could be visualized, and it was processed for hematoxylin and eosin staining on a Leica ST5020 Autostainer by the Ohio State University Comprehensive Cancer Center Comparative Pathology & Digital Imaging Shared Resource. The slides were then viewed at 100× and 400× with a Leica DMIL LED microscope and captured via an Excelis MPX-20C camera and CaptaVision+ Software v2.0.3.0 (Accu-Scope). Representative images were chosen from a slide with an average intensity infection from each group as approximated by white blood cell infiltration, typical skin structure disruption, and parasite observation. The 100× images were focused on the skin surface and deeper layers of the skin, while 400× images were focused on lesion tissue proximal to the epidermis within the lesion area. General qualitative trends in the tissue were compared, but no formal histological scoring was performed, which is a limitation.

### 2.14. Blood BUN and serum creatinine

The mouse blood urea nitrogen (BUN) and serum creatinine levels were employed as markers for kidney toxicity. The blood from the mice was first collected one day prior to the start of treatment, followed by one day after microneedle treatment ended. Assays were performed using the Vet Axcel Chemistry Analyzer (Alfa Wasserman) with serum samples per the manufacturer's instructions by the Ohio State University Comprehensive Cancer Center Comparative Pathology & Digital Imaging Shared Resource.

### 2.15. Flow cytometry

Single-cell suspensions from the organs of interest were plated at 1 × 10^6^–3 × 10^6^ cells/well in a 24-well plate in 1 mL RPMI/well. The remaining cells were mixed together to create the unstained control, single-stain control, and isotype control samples. All the wells were incubated for 4‒6 hours with Cell Activation Cocktail with Brefeldin A (BioLegend cat# 423303) at 37 °C so that cytokine production could be detected. The cells were then washed with PBS and stained (0.5 µL/1 × 10^6^ cells) for the following surface markers: CD3 (APC-anti Mouse CD3e, BioLegend cat# 100312), CD4 (BV650-anti Mouse CD4 Rat IgG2a, BioLegend cat# 100545), and CD8 (AF700-anti Mouse CD8a Rat IgG2a, BioLegend cat# 100730) in the presence of normal mouse serum to block nonspecific binding. Next, the cells were fixed in 4% paraformaldehyde, resuspended in PBS, and kept refrigerated overnight. Subsequently, the cells were permeabilized with 1x Intracellular Staining Perm Wash Buffer (BioLegend cat# 421002), and the intracellular cytokines and isotype controls for these antibodies were stained (1 µL per 1 × 10^6^ cells). The intracellular targets were IL-10 (PerCP/Cyanine 5.5-anti Mouse Rat IgG2a, BioLegend cat# 505028; PerCP/Cyanine 5.5 Rat IgG2a isotype control, BioLegend cat# 400631), IL-17A (BV510-anti Mouse Rat IgG1, BioLegend cat# 506933; BV510-Rat IgG1 isotype control, BioLegend cat# 400435), TNF-α (BV421-anti Mouse Rat IgG1, BioLegend cat# 506327; BV421-Rat IgG1 isotype control, BioLegend cat# 400429), IFN-γ (FITC-anti-Mouse Rat IgG1, BioLegend cat# 505806; FITC-Rat IgG1 isotype control, BioLegend cat# 400405), and IL-4 (PE-anti Mouse Rat IgG1, BioLegend cat# 504104; PE-Rat IgG1 isotype control, BioLegend cat# 400407). Flow cytometry was then performed with a FACSCelesta Flow Cytometer (BD Biosciences); analysis was undertaken using FlowJo software v10.0.9 (Tree Star, Inc., Ashland, OR, USA) using typical gating guidelines (Figure S2, Staats 2019).

### 2.16. Statistical analysis

Statistical analysis was performed in Anaconda Cloud Jupyter Notebook running Python v3.9.12, SciPy v1.11.4, and Pingouin v0.6.1. Statistical significance was calculated using the Mann‒Whitney U test; owing to limited sample sizes and, in some places, log-based data, non-normal distribution was assumed. No formal test was performed to verify this in each dataset, which is a limitation. These statistical tests were run with the ‘greater than’ hypothesis when the hypothesis tested involved directionality. However, when otherwise noted, two-tailed hypotheses were used if the objective was to test if there was a difference between groups. For all analyses, effect sizes were also reported via the area under the curve (AUC) method, a non-parametric measure that pairs with the Mann‒Whitney U test (Bao and Yi 2025). In our AUC tests, the placebo group is always Group 1, and the drug treatment group is always Group 2. If AUC = 0.5, there is no effect. AUC > 0.5, Placebo values > Drug values more often and vice versa for AUC < 0.5. The closer the value is to 0 or 1, the less overlap and the stronger the effects between the groups are. Additionally, *n* = 5 for all groups unless otherwise specified. A *p*-value < 0.05 was considered statistically significant. The error bars in all figures represent the standard error of the mean. Finally, denotions in figures are as follows: ***: *p*-value ≤ 0.005, **: *p*-value ≤ 0.050, *: 0.050 < *p*-value < 0.100 (when inconvenient to print full *p*-value), and n.s.: not significant.

## 3. Results

### 3.1. Penetration of hollow microneedles through the stratum corneum of the skin

**Figure 2.**
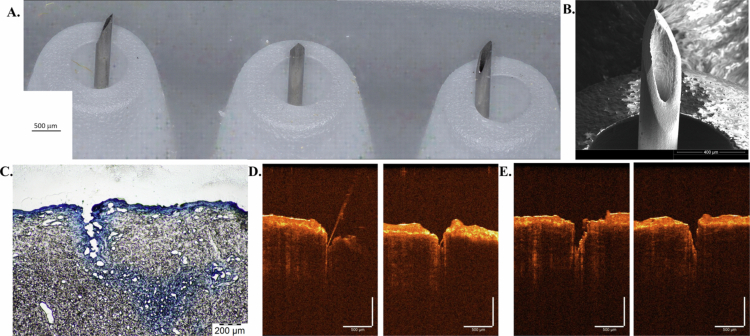
Characterization of hollow microneedle structure, skin penetration, and model drug diffusion. (A) Stitched optical representation of a single row of microneedles in the three-by-three hollow microneedle array. (B) Optical micrograph of a single microneedle located within the hollow microneedle array. (C) Light microscopy image showing methyl blue delivery in porcine skin that was pretreated with the microneedle array; methylene blue diffusion was observed in pig skin beyond the zone of penetration. Optical coherence tomography images of (D) microneedles inserted in porcine skin and (E) porcine skin after removal.

The microneedle devices were characterized in terms of their physical properties. Figure 2A shows a stitched optical representation of a single row of three microneedles in the three-by-three hollow microneedle array. This image confirms that the component needles exhibit a length of 1 mm and a pitch of 3.5 mm (Papich and Narayan 2022). Optical micrography was also performed on a single microneedle within the hollow microneedle array (Figure 2B). The image confirms that the inner diameter of the microneedle is 150 µm, the outer diameter of the microneedle is 250 µm, and the microneedle exhibits a sharp tip.

Porcine skin has been used to evaluate the penetration properties of microneedle arrays (Aldawood et al. 2021). A 1 mL syringe was used to preload methyl blue dye into the microneedles; the microneedles were subsequently inserted into the skin for injection. The microneedle-treated skin was then sectioned to observe the microneedle tracks. The cross-sections of the microneedle points were imaged using brightfield settings. The image exhibited a rupture of 464 µm depth into the skin, with approximately 1378 µm horizontal diffusion and 681 µm vertical diffusion within the skin (Figure 2C). The finding that the microneedle penetration depth in porcine skin was less than the microneedle length was attributed to the elastic properties of the skin. The depth of methyl blue dye penetration, as indicated by the brightfield image, demonstrated that microneedle array treatment delivered the model drug into the dermis with minimal damage.

Optical coherence tomography has been previously used to examine the penetration of microneedles into the skin (Enfield et al. 2010; Sattler et al. 2013). Optical coherence tomography images of microneedles inserted in porcine skin and porcine skin after removal of the microneedles are shown in Figures 2D, E, and S1. As previously noted by Enfield et al., the stratum corneum layer of the skin exhibits higher intensity than the underlying epidermal and dermal layers since it contains a high amount of keratin (Enfield et al. 2010). The image of the microneedles inserted in porcine skin indicated that the microneedles penetrated the stratum corneum layer of the skin; this skin layer serves as a barrier to the movement of many types of pharmaceutical agents. Prior studies have documented that the average thickness of the porcine stratum corneum is approximately 22 µm across various body parts (Krumpholz 2022); meanwhile, the needle penetration shown in Figure 2C is greater than 400 µm deep – a measurement that is consistent across multiple OCT imaging systems (Figure S1). The image of the porcine skin after the removal of the microneedles showed that the indentation in the skin from microneedle penetration was smaller than the microneedle height; this finding was attributed to the elastic properties of the skin.

### 3.2. CL lesion reduction under hollow microneedle Fungizone AmB treatment

**Figure 3.**
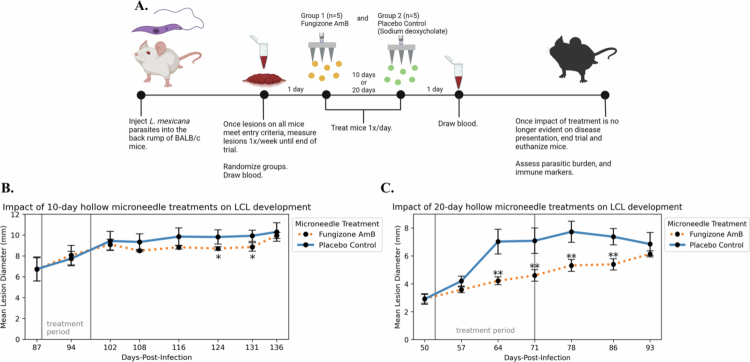
Experimental setup and clinical outcomes of hollow microneedle-based delivery of Fungizone Amphotericin B for cutaneous leishmaniasis. (A) Experimental setup: mice were infected, monitored, treated, and euthanized as described. Treatments were performed for 10 and 20 consecutive days in independent trials. (B) Lesion size over time following microneedle treatment for 10 days. The Fungizone AmB drug group did not achieve a significant difference relative to the placebo control treatment (minimum *p* = 0.075, AUC = 0.758, maximum size difference between groups was 1.1 mm). (C) Lesion size over time following treatment for 20 days. A significant difference was achieved at W2, which was maintained for weeks after cessation of treatment (minimum *p* = 0.008, AUC = 0.912, the maximum size difference between groups was 2.8 mm).

**Figure 4.**
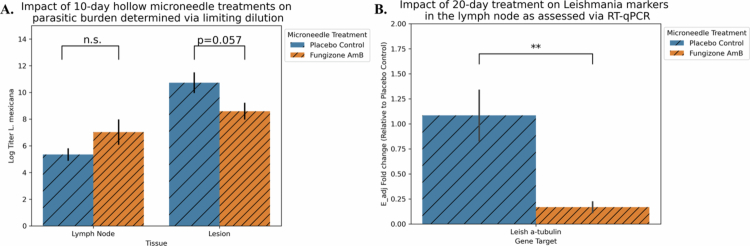
Microneedle-based delivery of Fungizone Amphotericin B reduces parasite burden, especially in the 20-day trial. (A) In the 10-day trial, parasitic burden was assessed via limiting dilution parasite growth. Lesion tissue from the placebo control group yielded a log titer of 2.1, about 100-fold more parasites than the lesion tissue from the microneedle-Fungizone AmB group (*p* = 0.057, AUC = 0.831). Meanwhile, lymph nodes from the 10-day trial had no significant difference (*p* = 0.290 via the Mann‒Whitney U test two-sided hypothesis, AUC = 0.238). (B) For the first 20-day trial, RT‒qPCR for the parasite gene α-tubulin showed a significant 0.17-fold change in parasite RNA from the lymph node tissue in the drug group relative to the placebo group (*p* = 0.018, AUC = 0.921). *n* = 5/group, except for the Fungizone-treated group (*n* = 3) in panel B, reflecting the inclusion of samples meeting melt curve quality criteria. E_adj = efficiency-adjusted.

To test our hypothesis regarding the potential benefits of microneedle-based delivery of Fungizone AmB directly to LCL lesions in BALB/c mice hosting *L. mexicana*, we performed three independent mouse experiments with identical infection protocols and exit criteria. Following lesion development, the groups in the first trial received 10 days of consecutive microneedle treatment while the groups in the second and third trials received 20 days of consecutive treatment (Figure 3A). The microneedle-based delivery of a placebo control solution containing the vehicle (deoxycholate) was used as a control. Lesion growth was measured weekly to observe the impact of microneedle Fungizone and placebo control delivery on the course of cutaneous lesion development. In the 10-day trial, there was a slight impact of the drug on slowing lesion development (not exceeding a 1.1 mm difference between the groups, *p* = 0.075, AUC = 0.758; Figure 3B). However, once treatment was elongated to 20 consecutive days, the hollow microneedle drug group had up to 2.8 mm smaller lesions on average compared to the placebo control group (for significant weeks, *p*-values ranged from 0.008–0.048, AUC = 0.867–0.912; Figure 3C). Similarly, in the repetition of the 20-day trial, lesion size was reduced in the Fungizone AmB group by up to 1.7 mm compared to the placebo control group (significant weeks *p* = 0.028, AUC = 0.849–0.863; Figure S3A). The reduction of lesion size correlates with parasitic burden data by both limiting dilution and qPCR methods (Figures 4 and S3B).

### 3.3. Reduction of parasite burden following microneedle delivery of Fungizone AmB

For the 10-day treatment, parasitic burden, as assessed by limiting dilution recovery and *in vitro* growth of viable parasites, was reduced in the lesion tissue of the drug microneedle group compared to the placebo control group (difference in log titer 2.1 or about 100-fold, *p* = 0.057, AUC = 0.831); meanwhile, we did not find a significant reduction in the lymph nodes (Figure 4A). In the first 20-day trial, parasitic burden was assessed by quantifying parasite transcript abundance. In the lymph node, the parasite burden of the microneedle Fungizone AmB treatment group was 0.17-fold greater than that of the microneedle placebo control group, as measured by *Leishmania* α-tubulin expression (*p* = 0.018, AUC = 0.921, Figure 4B). In the 20-day repetition trial, the limiting dilution assay was again performed, yielding an average reduction in parasite concentration from the lymph node tissue approaching 10^6^ (difference in log titer 5.8, *p* = 0.075, AUC = 0.757; Figure S3B). These data reinforce the efficacy of microneedle-based Fungizone AmB delivery for LCL treatment within each trial.

### 3.4. Altered tissue organization in the superficial skin of the Fungizone AmB treatment groups in histology

**Figure 5.**
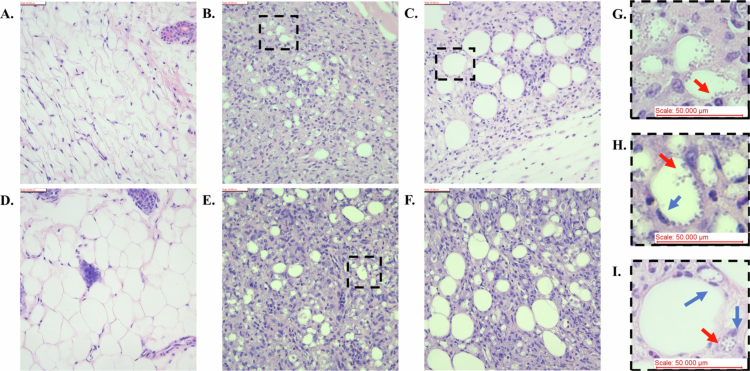
Microneedle-based delivery of Fungizone AmB over 10 and 20 days resulted in less disrupted superficial subepidermal tissue organization. These 400× histology images are from the skin lesion tissue of the 10-day microneedle trial (A–C, G, I) or 20-day repetition microneedle trial (D–F, H). All the images show adipose tissue and white blood cell infiltration proximal to the epidermis, and an average view of the group's appearance in this region of interest is shown. (A) Naïve (no treatment) mouse showing a healthy subepidermal adipose layer. (B) Placebo-treated mouse showing a highly infected subepidermal zone. (C) Fungizone AmB-treated mouse showing lesser *Leishmania* infection and white blood cell presence, and larger vacuole cells than seen in the placebo group. (D–F) As above, but from the 20-day treatment repetition trial. (G) A zoomed-in view of B to better show the *Leishmania* parasite marquee sign inside a large vacuole lacking a visible nucleus. (H). A zoomed-in view of E, showing a likely adipose cell as indicated by the small peripheral nucleus and with *Leishmania* amastigotes also visible. (I) A likely macrophage, as indicated by the larger nucleus-to-cytoplasm ratio, was filled with *L. mexicana* amastigotes. This panel originates from C. Denotation: Red arrows indicate *L. mexicana* amastigotes, blue arrows indicate host cell nuclei.

Adipose tissue lies below the stratum corneum and epidermis and typically shows little to no white blood cell infiltration and clear regions in naïve mice, as shown in both the 10-day trial and 20-day repetition (Figure 5A, D). However, in the experimental mice, heavy infection was observed, including intracellular *Leishmania* within the subepidermal vacuoles and white blood cell infiltration (Figure 5B). While observing the subepidermal layer within the lesion from Fungizone AmB mice (Fig 5C, 5F) in both the 10 and 20-day treatment trials, larger vacuoles are seen near the surface of the skin, with fewer parasites within them (many showing no infection), and less white blood cell infiltration was visible than in the placebo treatment group (Figure 5B, E). A zoomed-out view of the upper layers of the skin can be seen in Figure S4, which better indicates the large-scale differences in tissue between the drug and placebo groups. In particular, the lesions from the Fungizone AmB group (Figure S4C) show more typical superficial adipose tissue above and at the intersection of white blood cell infiltration than the placebo control group, which shows more dense white blood cell infiltration to the uppermost skin layer within the lesion (Figure S4B). The layers of the skin in the naïve mice are annotated in agreement with prior researchers for reference in Figure S4A (Smith et al. 2012). Additionally, individual parasites can be seen in both groups, as highlighted in Figure 5G; although this cell lacks an obvious nucleus in the cross-section. The infected cell types of interest are further highlighted in Figure 5H, which we hypothesize shows an infected adipocyte based on the nuclear morphology, and Figure 5I likely shows an infected macrophage neighboring an uninfected adipocyte.

### 3.5. Kidney toxicity markers were not observed during microneedle delivery of Fungizone AmB

**Figure 6.**
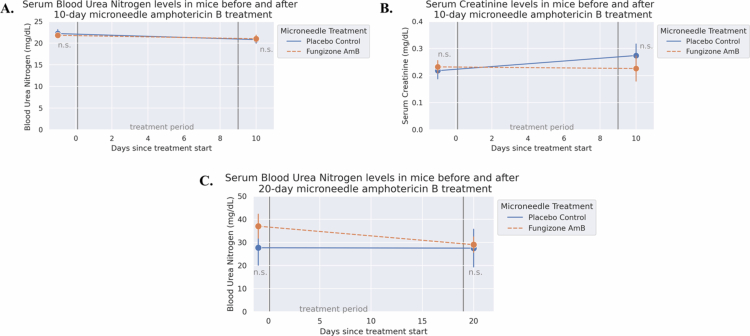
Microneedle-based delivery of Fungizone Amphotericin B had no impact on blood serum kidney toxicity markers following treatment. Serum samples collected one day before or after microneedle treatment periods were analyzed for kidney toxicity markers - blood urea nitrogen (BUN) and/or creatinine. *p*-values were assessed via the Mann‒Whitney U test with a two-sided hypothesis. (A) Serum BUN levels in the 10-day trial did not differ by any statistical significance between the treatment groups before or after treatment (*p* = 0.548, AUC = 0.575, and *p* = 0.690, AUC = 0.470, respectively). (B) Serum creatinine levels in the 10-day trial also did not differ by any statistical significance between the treatment groups (*p* = 0.841, AUC = 0.435, and *p* = 0.421, AUC = 0.633, respectively). (C) As in A, but for the 20-day treatment repetition trial. No statistically significant difference at either time point (*p* = 0.571, AUC = 0.293 and *p* = 0.686, AUC = 0.466, respectively). *n* = 5/group at all timepoints except for (C), which had *n* = 3 for placebo at day −1 and *n* = 4 for each group at day 20.

To evaluate kidney toxicity, blood samples from before and after treatment were evaluated for toxicity markers of blood urea nitrogen (BUN) and serum creatinine in the 10-day trial. We observed no kidney toxicity after the administration of Fungizone AmB via microneedles, as evidenced by no significant differences in either serum creatinine or BUN between the placebo and Fungizone AmB groups 1-day post-treatment (Figure 6A, B). There was also no observed elevation of serum BUN in the repetition of the 20-day treatment trial (Figure 6C).

### 3.6. Evaluating local and distal cytokine effects of Microneedle Fungizone AmB treatment for CL

**Figure 7.**
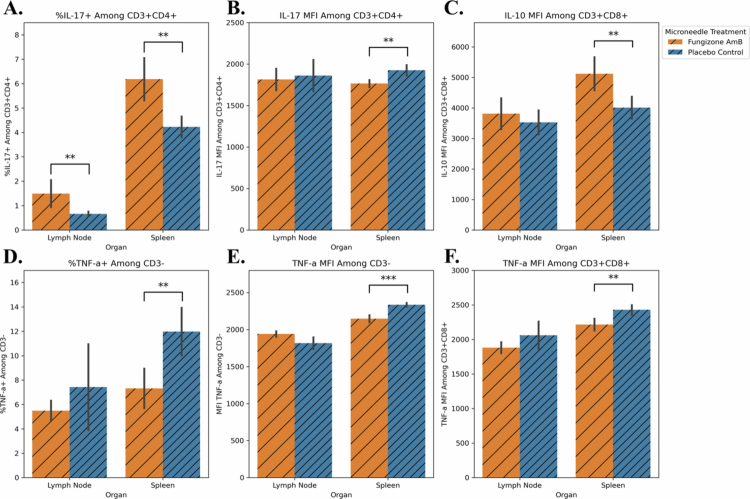
Microneedle-based delivery of Fungizone Amphotericin B over 10 days had few lasting local impacts on immune cell populations but more in the spleen. Cells obtained at the time of euthanasia for the 10-day trial originating from the lymph nodes and spleens of experimental and placebo control mice were assayed via flow cytometry for cytokine production. (A) The percent of CD3+CD4+IL-17+ cells was more abundant in the Fungizone AmB group for the lymph nodes (*p* = 0.048, AUC = 0.746) and spleen (*p* = 0.048, AUC = 0.818). (B) The mean fluorescence intensity (MFI) of the CD3+CD4+ cell population also had a more intense IL-17 signal in the spleen tissue from the placebo control over the Fungizone AmB group (*p* = 0.028, AUC = 0.827). (C) The MFI for IL-10 among the CD3+C8+ cells in the spleen was also higher in the Fungizone AmB group (*p* = 0.048, AUC = 0.779). (D) Meanwhile, the %CD3-TNF-α+ cells were significantly elevated in spleen tissue (*p* = 0.048, AUC = 0.794), and (E) TNF-α MFI among CD3- cells was elevated in the placebo control group (*p* = 0.004, AUC = 0.944), and the (F) percent of CD3+CD8+TNF-α+ cells was elevated in the placebo group also (*p* = 0.028, AUC = 0.805).

At the end of the 10-day trial, the cell population and cytokine expression in the draining lymph node and spleen tissues of the mice were explored via flow cytometry. The production of the cytokines TNF-α, IFN-γ, and IL-10 was scarcely affected by microneedle Fungizone AmB or placebo administration in the draining lymph nodes for all the cell populations assessed (CD3−, CD3+CD4+, and CD3+CD8+; data not shown). Statistically significant differences were, however, seen in the lymph node for the percentage of CD3+CD4+IL-17+ cells, which was elevated in the Fungizone AmB group (*p* = 0.048, AUC = 0.746), and this trend was also observed in the spleen groups with the same cell population (*p* = 0.048, AUC = 0.818, Figure 7A). Moreover, the data from the 10-day trial also suggest that IL-10 may be expressed at greater intensity in the Fungizone AmB group over the placebo among CD3+CD8+ cells from the spleen (*p* = 0.048, AUC = 0.779, Figure 7C). Furthermore, TNF-α appears elevated in the spleens of the placebo group (Figure 7D–F), including an elevated percentage of TNF-α+ and TNF-α mean fluorescence intensity (MFI) in cells in the CD3− spleen population (*p* = 0.048, AUC = 0.794 and *p* = 0.004, AUC = 0.944 respectively), and elevated TNF-α MFI in the CD3+CD8+ population (*p* = 0.028, AUC = 0.805).

**Figure 8.**
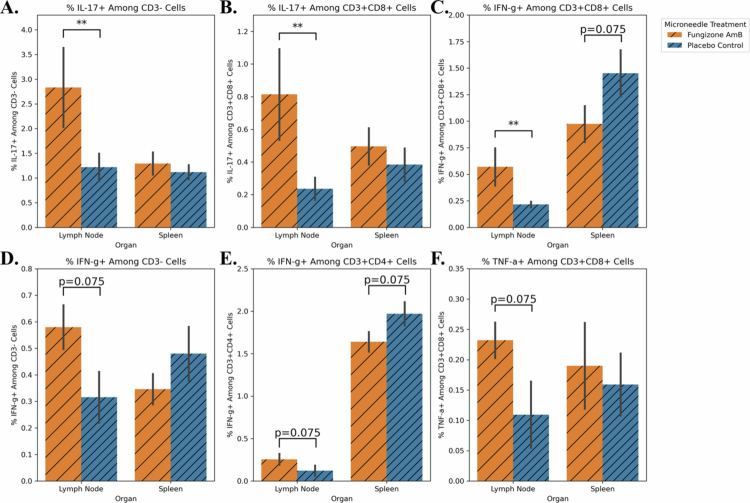
Microneedle treatment over 20 days significantly increased IL-17 and IFN-γ in the lymph node tissue of Fungizone-treated mice over placebo. Cells obtained at the time of euthanasia for the repetition of the 20-day trial originating from the lymph nodes and spleens of experimental and placebo control mice were characterized via flow cytometry for cytokine production. (A) A significant trend was observed in the lymph nodes of the Fungizone AmB group, which showed elevated percent CD3-IL-17+ cells (*p* = 0.016, AUC = 0.803). (B) A similar significantly elevated level of CD3+CD8+IL-17+ cells was detected in the Fungizone AmB LN group (*p* = 0.048, AUC = 0.817). (C) CD3+CD8+ cells also showed differences in the expression of IFN-γ. In this cell population, lymph nodes (*p* = 0.008, AUC = 0.814) showed higher levels in the drug group, and the spleen showed the inverse trend (*p* = 0.075, AUC = 0.782). (D) % CD3-IFN-γ+ cells also approached statistical significance for the Fungizone AmB group, having greater levels of these cells than the placebo group in the lymph node tissue (*p* = 0.075, AUC = 0.825). (E) % CD4+IFN-γ+ cells showed two inverse trends at each organ site. Lymph nodes had slightly higher levels of this cell type in the Fungizone AmB treatment group, whereas the spleen showed slight elevation for the placebo, and neither reached statistical significance (*p* = 0.075, AUC = 0.752 for LN and *p* = 0.075, AUC = 0.796 for spleen). (F) % CD3+CD8+TNF-α+ also showed elevated abundance in the Fungizone AmB-treated lymph nodes compared to the placebo (*p* = 0.075, AUC = 0.813).

At the end of the 20-day repetition trial, flow cytometry was repeated; some similar and some stronger effects were noted compared to the 10-day trial, especially in the lymph node tissue proximal to the CL lesion and treatment site. We again observed statistically significant upregulation of IL-17 in this lymph node tissue from the Fungizone AmB group compared to the placebo group for CD3− cells (Figure 8A, *p* = 0.016, AUC = 0.803) and for CD3+CD8+ cells (Figure 8B, *p* = 0.048, AUC = 0.817). IFN-γ also saw similar statistically significant upregulation along the same pattern for CD3+CD8+ cells (Figure 8C, *p* = 0.008, AUC = 0.814), and directional concordance was observed for CD3− and CD3+CD4+ cells of the lymph node (*p* = 0.075, AUC = 0.825, Figure 8D; *p* = 0.075, AUC = 0.752, Figure 8E). In contrast, the IFN-γ expression in the spleen showed some increase in the placebo group over the Fungizone AmB group for CD3+CD8+ and CD3+CD4+ cells (*p* = 0.075, AUC = 0.782, Figure 8C, *p* = 0.075, AUC = 0.796, Figure 8E). Furthermore, TNF-α was also more abundant in the Fungizone AmB lymph node CD3+CD8+ cells but did not achieve statistical significance (Figure 8F, *p* = 0.075, AUC = 0.813).

## 4. Discussion

As the range and prevalence of CL continue to rise (Vos et al. 2020), more effective and accessible treatments are needed. Current treatment varies by *Leishmania* species and location, but most anti-leishmanials used systemically pose high risks of toxicity; thus, localized therapies are preferred when possible (Aronson and Joya 2019; de Vries and Schallig 2022). Intralesional injection of the drug is one method to reduce toxicity and is recommended for treating LCL arising from *L. mexicana* (Aronson and Joya 2019). The antimonial compounds sodium stibogluconate and meglumine antimoniate are most utilized in this context; however, as resistance to antimonial drugs has risen, more options, such as intralesional AmB administration, are being explored (Goyonlo et al. 2014; Özbilgin et al. 2020). Topical treatments of paromomycin have also been developed for treatment, with encouraging results, proving that transcutaneous delivery can be effective for LCL (Ben Salah et al. 2013). AmB has also been studied in a formulation for topical application; while this was effective at reducing *L. major* lesion sizes, there was no change in lesions caused by *L. mexicana* (Layegh et al. 2011; Varikuti et al. 2017). The development of topical solutions is limited by the low bioavailability of drugs. As such, topical solutions often require surfactant supplementation or expensive liposomal encapsulation to aid penetration into oily skin (Shukla et al. 2022). Therefore, using microneedles for treatment could enhance the topical delivery of existing drugs in a more straightforward manner. Just as studies are evaluating microneedles to gather subcutaneous fluid samples (Samant et al. 2020) or to deliver *Leishmania* vaccines non-invasively (Moreno et al. 2017; Lanza et al. 2020), less-invasive microneedle treatments are also being tested for LCL. Previous studies have shown that the application of solid microneedles preceding a topical application of AmB or dissolvable microneedles loaded with AmB could also help reduce CL severity in mice, and similar applications have been investigated for cutaneous fungal infections (Nguyen et al. 2019; Zare et al. 2021; Peng et al. 2021; Dastan et al. 2025; Wang et al. 2025). However, our study represents the first positive result for the use of hollow, injectable microneedles for the treatment of LCL. These findings could improve topical AmB drug penetration while offering a less technical version of intralesional drug administration.

This study has thus shown clearly that hollow microneedles reliably pierce the stratum corneum and allow model drug injection directly into the epidermis of the skin (Figure 2). We have also shown that additional diffusion can occur deeper into the skin beyond the depth of the needle penetration (Figure 2C). Regarding the hollow microneedle Fungizone AmB treatments in mice, our three independent trials yielded similar trends. BALB/c mice cannot control *L. mexicana* as the lesions enlarge over time (Rosas et al. 2005); therefore, any alteration in lesion size is best explained by our experimental treatment. The 10- and 20-day treatment periods were chosen as these are within the maximum human patient treatment periods for IV Fungizone delivery (Rodríguez Galvis et al. 2020). Among these trials, a stronger effect was observed with respect to the LCL lesion size during the longer 20-day treatments, as evidenced by the earlier appearance of statistically significant differences and the larger magnitude of lesion size differences by group post-treatment compared to the 10-day trial (Figures 3, S3A). But, even during the 10-day treatment, the difference in lesion size approached significance (Figure 3B). Notably, although the AmB microneedle treatment slowed disease progression (lesion size), it did not lead to a clinical cure in any of the 3 trials. This could be due to an inadequate treatment period, drug penetration, drug dose, or the lack of protective immune responses, and the remaining parasites likely grew in number again after treatment ended. We further demonstrated that hollow microneedle-based injection of Fungizone AmB reduced parasitic burden markers within each trial, including viable parasite recovery and parasite gene expression levels, following both 10-day and 20-day daily treatment periods (Figures 4, S3B). However, as a shorter treatment period would be desirable to improve patient compliance, hollow microneedles could be further optimized with this goal in mind. For example, several experimental single-dose CL treatments have been attempted via the injection of novel AmB formulas in mice (Sousa-Batista et al. 2018a, 2018b, 2019, 2020) and pentamidine in humans (Gadelha et al. 2015, 2018). Notably, multiple studies have shown that AmB-resistant lines become hypersensitive to pentamidine treatment (Mwenechanya et al. 2017; Pountain et al. 2019). Therefore, future microneedle studies involving AmB and pentamidine in combination could be an attractive next step.

Our study also revealed a distinction between the Fungizone AmB treatment group and the placebo control group in the appearance of the superficial layers of the dermis via histology. In both groups, large lesion sections containing white blood cell infiltration and parasite-infected cells were observed at the time of euthanasia, several weeks' post-treatment. However, in the Fungizone AmB group from both the 10- and 20-day trials, larger vacuolated cells appeared preserved, and some did not contain parasites (Figures 5C, F, S4C). This finding was more similar to the state of the naïve adipose tissue in the upper layers of the skin (Figures 5A, D, S4A). In contrast, the same upper region of the skin lesion showed much smaller infected vacuoles in the placebo control group, with more white blood cell infiltration throughout the upper layers of the dermis (Figures 5B, E, S4B). These observations could indicate that the Fungizone AmB delivery via the microneedle was most effective at superficial depths of the lesion; further testing may be needed to show or improve efficacy in deeper tissue.

Moreover, prior researchers have observed that *Leishmania* amastigotes in histology residing at the edges of distinctive, enlarged parasitophorous vacuoles within the upper layers of the skin during cutaneous leishmaniasis and dubbed them the characteristic ‘marquee sign.’ Many studies report that these vacuoles are within macrophages (Grimaldi et al. 1980, 1984; Biddlestone et al. 1994); however, in our study, it was sometimes difficult to discern whether the vacuole-containing cells were macrophages or adipocytes, especially if the nucleus of the vacuolated cell was not captured by the tissue section that was stained. Mature intradermal, dermal, and subcutaneous white adipose tissue tends to have marginal nuclei, which could be one method to distinguish the cell type (Driskell et al. 2014). Recent authors are exploring the mechanisms of *Leishmania* entry into non-phagocytic cell types, such as adipocytes, and this phenomenon is supported by evidence of adipocyte infection by *Leishmania infantum, Leishmania braziliensis, and L. amazonensis* (Schwing et al. 2021; Mendes et al. 2022; Valigurová and Kolářová 2023). Notably, *L. mexicana* is of the same subgenus as *L. infantum* and *L. amazonensis* (Waki et al. 2007). Thus, especially in marginal areas (periphery and microneedle-treated surface regions) of the lesion where adipocyte organization remains relatively intact, *L. mexicana* adipocyte infection is worth considering as a possibility. Figure 5H could be the first evidence of *L. mexicana* adipocyte-attributed infection, but further verification is needed.

While the microneedles showed efficacy in reducing parasitic burden and preserving more typical skin architecture near the surface, we observed no short-term toxicity from this route of Fungizone AmB administration in the 10-day and 20-day trials. Specifically, treatment did not affect the levels of the kidney toxicity markers BUN or serum creatinine in the blood immediately after the end of treatment (Figure 6). In contrast, previous studies have shown that IV administration of a similar or equivalent dose of 1 mg/kg/day Fungizone AmB is sufficient to observe elevated levels of these renal toxicity markers after only 1–3 days of treatment (Hossain et al. 2000; Khan and Owais 2006), although a direct comparison study would better confirm this distinction. Moreover, one recent study showed that dissolving microneedles resulted in highly localized delivery with little detectable AmB in the liver, spleen, and plasma (Wang et al. 2025). Future studies could investigate such pharmacokinetic information in our system, as we used a different drug doses and formulas, and further verify the absence of liver in addition to kidney toxicity markers.

Regarding immune differences between the drug and placebo groups, we decided to analyze targets that were previously known to impact the course of *L. mexicana* or the closely related *Leishmania amazonensis* infections. IL-4 and IL-10 are known to be anti-inflammatory and can be counterproductive for parasite clearance (Padigel et al. 2003; Guimarães et al. 2006; Buxbaum 2015), IFN-γ is known to be pro-inflammatory and generally productive (Chan 1993; Guevara-Mendoza et al. 1997), IL-17 and TNF-α are both pro-inflammatory, with more mixed impacts or potentially harmful impacts (Bacellar et al. 2009; Sousa et al. 2014; González-Tafoya et al. 2020), and CD4+ and CD8+ T cells are known to be critical for protection in part due to their ability to produce or induce these different cytokines. Also, TNF-α is among the list of cytokines that can be induced by Amphotericin B toxicity, especially at the site of injection (Hamill 2013).

For these exploratory immune markers in the 10-day trial, there were few lasting, localized impacts on the immune profile, especially in the lymph nodes (Figure 7). Specifically, a statistically significant increase was seen in the percentage of CD3+CD4+IL-17+ cells in the draining lymph nodes of the microneedle Fungizone AmB group (Figure 7A). Meanwhile, in the 20-day trial, statistically significant increases in IL-17+ cell population were also observed in the lymph nodes of the Fungizone AmB-treated mice, specifically for CD3- and CD3+CD8+ cells (Figure 8A, B). Prior studies have shown that the elevated levels of CD4+IL-17+ cells co-occur with elevated neutrophil recruitment and disease perpetuation during *L. mexicana* infection in BALB/c mice (Pedraza-Zamora et al. 2017). Also, in other disease contexts, IL-17 can recruit neutrophils and induce neutrophil extracellular trap formation, which in turn further upregulates IL-17 production (Zhang et al. 2020; Kim et al. 2023). Thus, an AmB-driven excess of inflammatory IL-17 – which we observed in both the 10- and 20-day treatment trials – could increase arginase levels in host cells, as seen by Sousa et al. (2014), supporting parasite recovery during incomplete clearance. This altered inflammation and parasite recovery could explain why, although we observed significantly fewer parasites in the lesion tissue at our endpoint, the sizes of the lesions were comparable at the conclusion of the 10- and 20-day trials. In addition, the 20-day trial saw statistically significant increases in the abundance of IFN-γ+ cells in the lymph nodes of Fungizone AmB-treated mice, with CD3+CD8+ cells being most strongly affected, and a similar directional trend in CD3- and CD3+CD4+ cells (Figure 8C–E). Because IFN-γ is critical for control of *L. mexicana*, it could act synergistically with AmB-direct killing of parasites, leading to a reduced parasite load (Mendes et al. 2017; Virginia Tech 2017). Finally, TNF-α was elevated in CD3+CD8+ cells from the 20-day trial for lymph node tissue from the Fungizone AmB group (Figure 8F). TNF-α has been found previously to induce T-cell exhaustion and depress the production of pro-inflammatory cytokines, thus favoring *L. mexicana* persistence (Guimarães et al. 2006).

Furthermore, multiple observations of immune differences were explored in the spleen. The BALB/c mouse can have *L. mexicana* visceralize and infect the spleen at relatively low levels (Torrentera et al. 2002; Abdala et al. 2002; Pedraza-Zamora et al. 2017); thus, immune cell differences in the spleen could result from the parasite infection, from systemic cytokine levels, or, less likely, due to a low dose of drug diffusion into the spleen, potentially similar to the previously mentioned pharmacokinetic microneedle AmB findings (Wang et al. 2025). Regarding specific splenic changes, from the 10-day trial (Figure 7), IL-10 MFI was elevated in CD3+CD8+ splenic cells from the Fungizone AmB group, which could be indicative of CD8+ Tregs, pending further confirmation (Mishra et al. 2021). However, prior work revealed that IL-10 from T cell populations, but not the CD25+ Treg population, is necessary for perpetuating chronic *L. mexicana* infection (Thomas and Buxbaum 2008; Buxbaum 2015). Therefore, IL-10 upregulation in our Fungizone AmB group could also contribute to parasite persistence following treatment. Finally, TNF-α was also upregulated in multiple cell populations in our placebo group relative to the Fungizone AmB group (Figure 7D–F). Whereas, in the 20-day trial, the spleen did not reveal any statistically significant differences between the groups, but some trends were seen in IFN-γ. More CD3+CD4+ and CD3+CD8+ cells were found to express IFN-γ in the placebo group than in the Fungizone AmB group (Figure 8C, F), which contrasts with our lymph node tissue observations. IFN-γ is systemically produced in the spleen throughout *L. mexicana* infection at low levels in a BALB/c mouse model (Guevara-Mendoza et al. 1997; Pérez-Santos and Talamás-Rohana 2001), but why exactly any of these cytokines are expressed in one group over the other is unclear. More work, including immune evaluation at earlier timepoints, is needed to confirm these exploratory differences observed and whether they are due to Fungizone AmB administration, decreased parasite levels, or other elements in the immune system.

Additional investigations should also be performed to determine whether hollow, injectable needles may result in deeper penetration of the drug compared to alternative solid and dissolvable options, which were previously investigated (Nguyen et al. 2019; Zare et al. 2021; Peng et al. 2021; Dastan et al. 2025; Wang et al. 2025). Further research should also explore whether the hollow design results in similar or improved efficacy compared to the current standards of IV administration of Fungizone AmB, L-AmB, or other experimental CL treatments. Potential synergy as a companion to IV treatments should also be evaluated. Ultimately, hollow microneedles could one day be tested in human patients for their efficacy and practicality. Regarding cost, the materials for the microneedles are inexpensive (polypropylene body and stainless-steel needles joined by epoxy), and we repurposed the inexpensive Fungizone AmB drug formulation in this trial. After additional testing, at-home microneedle treatment could further reduce patients' economic burden from seeking CL treatment by reducing hospital stay length if at-home microneedle treatment achieves parity with current standard methods. Owing to ease of use, this could be comparable to how diabetic patients are trained to self-monitor their blood glucose levels and administer their own insulin injections (Dovc and Battelino 2020). Finally, we discussed how L-AmB IV treatment faces barriers to use in remote areas due to a lack of cold-chain transportation and storage in tropical environments, which are endemic for CL. Meanwhile, based on the polypropylene, epoxy, and stainless-steel components of the hollow microneedles, these devices can be assumed to be thermally stable even at elevated tropical temperatures that are expected during transportation and storage.

## 5. Conclusions

Overall, these results provide proof of concept that hollow microneedles are a promising treatment method for LCL in the context of *L. mexicana* infection. More work is needed to demonstrate whether this promise extends to improving clinical outcomes with Fungizone AmB, especially in direct comparison with standard of care treatments such as Fungizone AmB IV and L-AmB IV administration, with larger blinded studies, additional safety characterization of microneedle delivery, and pharmacokinetic studies. However, the potential for microneedles to improve treatment accessibility for patients is promising. In addition to being an easier means of administration for patients and healthcare providers, our exploratory results show that the use of microneedles may also avoid kidney toxicity issues reported previously with other routes of Fungizone AmB delivery. Using microneedles instead of a liposomal solution to prevent toxicity would be more feasible for many remote areas where CL is found. Furthermore, the non-technical nature of microneedle administration could reduce hospital stays, as patients could bring their treatment home with them for self-administration to ease the financial burden of leishmaniasis treatment, improve patient compliance, and therefore reduce parasite transmission. While these results are hopeful, more work will have to be done to investigate whether these results translate to humans and additional contexts in which these novel hollow microneedles may be useful.

## Supplementary Material

Supplementary Material Microneedle_ARRIVE_Checklist_20260411.pdf

Supplementary Material Supplemental_Online_Material.docx

## Data Availability

The data that support the findings of this study are available from ARS upon reasonable request.

## References

[cit0001] Abdala H, Alvarez N, Delmas F, et al. 2002. In vitro and in vivo antileishmanial activity of 2-amino-4, 6-dimethylpyridine derivatives against *Leishmania mexicana*. Parasite. 9(4):367–370. 10.1051/parasite/2002094367 12514953

[cit0002] Abd-El-Azim H, Tekko IA, Ali A, et al. 2022. Hollow microneedle assisted intradermal delivery of hypericin lipid nanocapsules with light enabled photodynamic therapy against skin cancer. J Control Release. 348:849–869. 10.1016/j.jconrel.2022.06.027 35728715

[cit0003] Aghakhani N, Azami M, Mohaghegh MA. 2023. Cutaneous leishmaniasis lowers the quality of life: a neglected truth. GMS Hyg Infect Control. 18. 10.3205/dgkh000447 PMC10566011 37829252

[cit0004] Aldawood FK, Andar A, Desai S. 2021. A comprehensive review of microneedles: types, materials, processes, characterizations and applications. Polymers. 13(16):2815. 10.3390/polym13162815 34451353 PMC8400269

[cit0005] Al-Mohammed HI, Chance ML, Bates PA. 2005. Production and characterization of stable amphotericin-resistant amastigotes and promastigotes of *Leishmania mexicana*. Antimicrob Agents Chemother. 49(8):3274–3280. 10.1128/AAC.49.8.3274-3280.2005 16048936 PMC1196255

[cit0006] Alpizar-Sosa EA, Ithnin NRB, Wei W, et al. 2022. Amphotericin B resistance in *Leishmania mexicana*: alterations to sterol metabolism and oxidative stress response. PLoS Negl Trop Dis. 16(9):e0010779. 10.1371/journal.pntd.0010779 36170238 PMC9581426

[cit0007] Aronson NE, Joya CA. 2019. Cutaneous leishmaniasis updates in diagnosis and management. Infect Dis Clin North Am. 33(1):101–117. 10.1016/j.idc.2018.10.004 30712756

[cit0008] Aronson N, Herwaldt BL, Libman M, et al. 2016. Diagnosis and treatment of leishmaniasis: clinical practice guidelines by the Infectious Diseases Society of America (IDSA) and the American society of tropical Medicine and hygiene (ASTMH). Clin Infect Dis. 63(12):1539–1557. 10.1093/cid/ciw742 27941143

[cit0009] Arya J, Henry S, Kalluri H, et al. 2017. Tolerability, usability and acceptability of dissolving microneedle patch administration in human subjects. Biomaterials. 128:1–7. 10.1016/j.biomaterials.2017.02.040 28285193 PMC5382793

[cit0010] Bacellar O, Faria D, Nascimento M, et al. 2009. IL-17 production in patients with American cutaneous leishmaniasis. J Infect Dis. 200(1):75–78. 10.1086/599380 19476435 PMC2732405

[cit0011] Bailey F, Mondragon-Shem K, Hotez P, et al. 2017. A new perspective on cutaneous leishmaniasis—Implications for global prevalence and burden of disease estimates. PLoS Negl Trop Dis. 11(8):e0005739. 10.1371/journal.pntd.0005739 28796782 PMC5552022

[cit0012] Bao Z, Yi B. 2025. Analysis of preoperative serum cytokine levels in patients with oral squamous cell carcinoma. Sci Rep. 15(1), 10.1038/s41598-025-89816-1 PMC12009433 40253468

[cit0013] Bates DW, Su L, Yu DT, et al. 2001. Mortality and costs of acute renal failure associated with amphotericin B therapy. Clin Infect Dis. 32(5):686–693. 10.1086/319211 11229835

[cit0014] Ben Salah A, Ben Messaoud N, Guedri E, et al. 2013. Topical paromomycin with or without gentamicin for cutaneous leishmaniasis. NEJM. 368(6):524–532. 10.1056/NEJMoa1202657 23388004

[cit0015] Bennis I, De Brouwere V, Belrhiti Z, et al. 2018. Psychosocial burden of localised cutaneous leishmaniasis: a scoping review. BMC Public Health. 18(1):358. 10.1186/s12889-018-5260-9 29544463 PMC5855994

[cit0016] Biddlestone LR, Hepburn NC, McLaren KM. 1994. A clinico-pathological study of cutaneous leishmaniasis in British troops from Belize. Trans R Soc Trop Med Hyg. 88(6):672–676. 10.1016/0035-9203(94)90223-2 7533953

[cit0017] Boukthir A, Bettaieb J, Erber AC, et al. 2020. Psycho-social impacts, experiences and perspectives of patients with cutaneous leishmaniasis regarding treatment options and case management: an exploratory qualitative study in Tunisia. PLoS One. 15(12):e0242494. 10.1371/journal.pone.0242494 33259489 PMC7707605

[cit0018] Bustin SA, Ruijter JM, van den Hoff MJB, et al. 2025. MIQE 2.0: revision of the minimum information for publication of quantitative real-time PCR experiments guidelines. Clin Chem. 71(6):634–651. 10.1093/clinchem/hvaf043 40272429

[cit0019] Buxbaum LU. 2015. Interleukin-10 from T cells, but not macrophages and granulocytes, is required for chronic disease in *Leishmania mexicana* infection. Infect Immun. 83(4):1366–1371. 10.1128/IAI.02909-14 25605773 PMC4363430

[cit0020] Chan MM. 1993. T cell response in murine *Leishmania mexicana* amazonensis infection: production of interferon-gamma by CD8+ cells. Eur J Immunol. 23(5):1181–1184. 10.1002/eji.1830230532 8097471

[cit0021] Chen X, Wang L, Yu H, et al. 2018. Preparation, properties and challenges of the microneedles-based insulin delivery system. J Control Release. 288:173–188. 10.1016/j.jconrel.2018.08.042 30189223

[cit0022] Croft SL, Sundar S, Fairlamb AH. 2006. Drug resistance in leishmaniasis. Clin Microbiol Rev. 19(1):111–126. 10.1128/CMR.19.1.111-126.2006 16418526 PMC1360270

[cit0023] Dar MJ, Khalid S, McElroy CA, et al. 2020. Topical treatment of cutaneous leishmaniasis with novel amphotericin B-miltefosine co-incorporated second generation ultra-deformable liposomes. Int J Pharm. 573:118900. 10.1016/j.ijpharm.2019.118900 31765786

[cit0024] Dastan N, Maghsood AH, Hatam G, et al. 2025. Experimental evaluation of the therapeutic effect of niosomal miltefosine delivered via polyvinylpyrrolidone - hyaluronic acid microneedle patches against *Leishmania major*. Int J Biiol Macromol. 334(Pt 2):149139. 10.1016/j.ijbiomac.2025.149139 41265603

[cit0025] Dovc K, Battelino T. 2020. Evolution of diabetes technology. Endocrinol Metab Clin North Am. 49(1):1–18. 10.1016/j.ecl.2019.10.009 31980111

[cit0026] Driskell R, Jahoda CAB, Chuong C, et al. 2014. Defining dermal adipose tissue. Exp Dermatol. 23(9):629–631. 10.1111/exd.12450 24841073 PMC4282701

[cit0027] Dul M, Alali M, Ameri M, et al. 2023. Assessing the risk of a clinically significant infection from a microneedle array patch (MAP) product. J Control Release. 361:236–245. 10.1016/j.jconrel.2023.07.001 37437849

[cit0028] Enfield J, O'Connell M, Lawlor K, et al. 2010. In-vivo dynamic characterization of microneedle skin penetration using optical coherence tomography. JBO. 15(4):046001. 10.1117/1.3463002 20799803

[cit0029] Fernández-García R, Statts L, de Jesus JA, et al. 2020. Ultradeformable lipid vesicles localize amphotericin B in the dermis for the treatment of infectious skin diseases. ACS Infect Dis. 6(10):2647–2660. 10.1021/acsinfecdis.0c00293 32810398

[cit0030] Fong D, Wallach M, Keithly J, et al. 1984. Differential expression of mRNAs for alpha- and beta-tubulin during differentiation of the parasitic protozoan *Leishmania mexicana*. Proc Nat Acad Sci USA. 81(18):5782–5786. 10.1073/pnas.81.18.5782 6592587 PMC391795

[cit0031] Gadelha EPN, Talhari S, Guerra JAdO, et al. 2015. Efficacy and safety of a single dose pentamidine (7mg/kg) for patients with cutaneous leishmaniasis caused by L. Guyanensis: a pilot study. An Bras Dermatol. 90(6):807–813. 10.1590/abd1806-4841.20153956 26734860 PMC4689067

[cit0032] Gadelha EPN, Ramasawmy R, da Costa Oliveira B, et al. 2018. An open label randomized clinical trial comparing the safety and effectiveness of one, two or three weekly pentamidine isethionate doses (seven milligrams per kilogram) in the treatment of cutaneous leishmaniasis in the Amazon region. PLoS Negl Trop Dis. 12(10):e0006850. 10.1371/journal.pntd.0006850 30379814 PMC6231690

[cit0033] Ghotloo S et al. 2015. Comparison of parasite burden using real-time polymerase chain reaction assay and limiting dilution assay in *L**eishmania major* infected mouse. Iran J Parasitol. 10(4):571. 26811723 PMC4724833

[cit0034] González-Tafoya E, Diupotex M, Zamora‐Chimal J, et al. 2020. TNF contributes to T-cell exhaustion in chronic L. Mexicana infections of mice through PD-L1 up-regulation. Cell Immunol. 358:104196. 10.1016/j.cellimm.2020.104196 33032241

[cit0035] Goyonlo VM, Vosoughi E, Kiafar B, et al. 2014. Efficacy of intralesional amphotericin B for the treatment of cutaneous leishmaniasis. Indian J Dermatol. 59(6):631–631. 10.4103/0019-5154.143571 PMC4248523 25484415

[cit0036] Grimaldi G, Moriearty PL, Hoff R. 1980. Leishmania mexicana: immunology and histopathology in C3H mice. Exp Parasitol. 50(1):45–56. 10.1016/0014-4894(80)90006-5 7389857

[cit0037] Grimaldi G, Soares MJ, Moriearty PL. 1984. Tissue eosinophilia and *Leishmania mexicana* mexicana eosinophil interactions in murine cutaneous leishmaniasis. Parasite Immunol. 6(5):397–408. 10.1111/j.1365-3024.1984.tb00811.x 6504555

[cit0038] Guevara-Mendoza O, Guevara‐mendoza O, Une C, et al. 1997. Experimental infection of balb/c mice with *Leishmania panamensis* and *Leishmania mexicana*: induction of early IFN-γ but not IL-4 is associated with the development of cutaneous lesions. Scand J Immunol. 46(1):35–40. 10.1046/j.1365-3083.1997.d01-96.x 9246206

[cit0039] Guimarães ET, Santos LA, Ribeiro dos Santos R, et al. 2006. Role of interleukin-4 and prostaglandin E2 in *Leishmania amazonensis* infection of BALB/c mice. Microbes Infect. 8(5):1219–1226. 10.1016/j.micinf.2005.11.011 16531090

[cit0040] Gupta J, Park SS, Bondy B, et al. 2011. Infusion pressure and pain during microneedle injection into skin of human subjects. Biomaterials. 32(28):6823–6831. 10.1016/j.biomaterials.2011.05.061 21684001 PMC3143217

[cit0041] Haghdoust S, Azizi M, Haji Molla Hoseini M, et al. 2020. Parasite burden measurement in the *Leishmania major* infected mice by using the direct fluorescent microscopy, limiting dilution assay, and real-time PCR analysis. Iran J Parasitol. 15(4):576. 10.18502/ijpa.v15i4.4867 33884015 PMC8039490

[cit0042] Hamill RJ. 2013. Amphotericin B formulations: a comparative review of efficacy and toxicity. Drugs. 73(9):919–934. 10.1007/s40265-013-0069-4 23729001

[cit0043] Handler MZ, Patel PA, Kapila R, et al. 2015. Cutaneous and mucocutaneous leishmaniasis: differential diagnosis, diagnosis, histopathology, and management. J Am Acad Dermatol. 73(6):911–926. 10.1016/j.jaad.2014.09.014 26568336

[cit0044] Hossain MA et al. 2000. Attenuation of nephrotoxicity by a novel lipid nanosphere (NS-718) incorporating amphotericin B. J Antimicrob Chemother. 46(2):263–268. 10.1093/jac/46.2.263 10933650

[cit0045] Hotez PJ, Remme JH, Buss P, et al. 2004. Combating tropical infectious diseases: report of the disease control priorities in developing countries project. Clin Infect Dis. 38(6):871–878. 10.1086/382077 14999633

[cit0046] Hultström M, Roxhed N, Nordquist L. 2014. Intradermal insulin delivery: a promising future for diabetes management. J Diabetes Sci Technol. 8(3):453–457. 10.1177/1932296814530060 24876605 PMC4455430

[cit0047] Ingrole RSJ, Azizoglu E, Dul M, et al. 2021. Trends of microneedle technology in the scientific literature, patents, clinical trials and Internet activity. Biomaterials. 267:120491. 10.1016/j.biomaterials.2020.120491 33217629 PMC8042615

[cit0048] Khan MA, Owais M. 2006. Toxicity, stability and pharmacokinetics of amphotericin B in immunomodulator tuftsin-bearing liposomes in a murine model. J Antimicrob Chemother. 58(1):125–132. 10.1093/jac/dkl177 16709592

[cit0049] Kim TS, Silva LM, Theofilou VI, et al. 2023. Neutrophil extracellular traps and extracellular histones potentiate IL-17 inflammation in periodontitis. J Exp Med. 220(9). 10.1084/jem.20221751 PMC10236943 37261457

[cit0050] Krumpholz L. 2022. Pig skin anatomy and physiology. Mendeley Data. 1. 10.17632/mwz9xv4cpd.1 PMC9547536 36208224

[cit0051] Lafuse WP, Story R, Mahylis J, et al. 2013. *Leishmania donovani* infection induces anemia in hamsters by differentially altering erythropoiesis in bone marrow and spleen. PLoS One. 8(3):e59509. 10.1371/journal.pone.0059509 23533629 PMC3606219

[cit0052] Lanza JS, Vucen S, Flynn O, et al. 2020. A TLR9-adjuvanted vaccine formulated into dissolvable microneedle patches or cationic liposomes protects against leishmaniasis after skin or subcutaneous immunization. Int J Pharm. 586:119390. 10.1016/j.ijpharm.2020.119390 32540349

[cit0053] Layegh P et al. 2011. Efficacy of topical liposomal amphotericin B versus intralesional meglumine antimoniate (Glucantime) in the treatment of cutaneous leishmaniasis. J Parasitol Res. 2011:656523. 22174993 10.1155/2011/656523 PMC3228299

[cit0054] McGwire BS, Satoskar AR. 2014. Leishmaniasis: clinical syndromes and treatment. QJM. 107(1):7–14. 10.1093/qjmed/hct116 23744570 PMC3869292

[cit0055] McIlwee BE, Weis SE, Hosler GA. 2018. Incidence of endemic human cutaneous leishmaniasis in the United States. JAMA dermatology. 154(9):1032. 10.1001/jamadermatol.2018.2133 30046836 PMC6143046

[cit0056] Mendes B, Minori K, Consonni SR, et al. 2022. Causative agents of American tegumentary leishmaniasis are able to infect 3T3-L1 adipocytes in vitro. Front Cell Infect Microbiol. 12. 10.3389/fcimb.2022.824494 PMC8855065 35186797

[cit0057] Mishra S, Srinivasan S, Ma C, et al. 2021. CD8+ regulatory T cell – A mystery to be revealed. Front Immunol. 12. 10.3389/fimmu.2021.708874 PMC8416339 34484208

[cit0058] Monroy-Ostria A, Sanchez-Tejeda G. 2017. Survey of cutaneous leishmaniasis in Mexico: leishmania species, clinical expressions and risk factors. The Epidemiology and Ecology of Leishmaniasis. IntechOpen. 10.5772/65501

[cit0059] Monteiro-Riviere NA, Bristol DG, Manning TO, et al. 1990. Interspecies and interregional analysis of the comparative histologic thickness and laser Doppler blood flow measurements at five cutaneous sites in nine species. J Invest Dermatol. 95(5):582–586. 10.1111/1523-1747.ep12505567 2230221

[cit0060] Moreno E, Schwartz J, Calvo A, et al. 2017. Skin vaccination using microneedles coated with a plasmid DNA cocktail encoding nucleosomal histones of leishmania spp. Int J Pharm. 533(1):236–244. 10.1016/j.ijpharm.2017.09.055 28964902

[cit0061] Mwenechanya R, Kovářová J, Dickens NJ, et al. 2017. Sterol 14α-demethylase mutation leads to amphotericin B resistance in *Leishmania mexicana*. PLoS Negl Trop Dis. 11(6):e0005649. 10.1371/journal.pntd.0005649 28622334 PMC5498063

[cit0062] Nguyen AK, Yang K, Bryant K, et al. 2019. Microneedle-based delivery of amphotericin B for treatment of cutaneous leishmaniasis. Biomed Microdevices. 21(1):8. 10.1007/s10544-018-0355-8 30617619 PMC10357955

[cit0063] Okwor I, Uzonna J. 2016. Social and economic burden of human leishmaniasis. Am J Trop Med Hyg. 94(3):489–493. 10.4269/ajtmh.15-0408 26787156 PMC4775878

[cit0064] Özbilgin A, Çavuş İ, Kaya T, et al. 2020. Comparison of in vitro resistance of wild leishmania İsolates, which are resistant to pentavalent antimonial compounds, against drugs used in the treatment of leishmaniasis. Turkiye Parazitolojii Dergisi. 44(1):12–16. 10.4274/tpd.galenos.2019.6661 32212583

[cit0065] Padigel UM, Alexander J, Farrell JP. 2003. The role of Interleukin-10 in susceptibility of BALB/c mice to infection with *Leishmania mexicana* and *Leishmania amazonensis*. J Immunol. 171(7):3705–3710. 10.4049/jimmunol.171.7.3705 14500669

[cit0066] Papich MG, Narayan RJ. 2022. Naloxone and nalmefene absorption delivered by hollow microneedles compared to intramuscular injection. Drug Deliv Transl Res. 12(2):376–383. 10.1007/s13346-021-01096-0 34817831 PMC10703510

[cit0067] Pedraza-Zamora CP, Pedraza‐Zamora CP, Delgado‐Domínguez J, et al. 2017. Th17 cells and neutrophils: close collaborators in chronic *Leishmania mexicana* infections leading to disease severity. Parasite Immunol. 39(4). 10.1111/pim.12420 28207936

[cit0068] Peng K, Vora LK, Tekko IA, et al. 2021. Dissolving microneedle patches loaded with amphotericin B microparticles for localised and sustained intradermal delivery: potential for enhanced treatment of cutaneous fungal infections. J Control Release. 339:361–380. 10.1016/j.jconrel.2021.10.001 34619227

[cit0069] Pérez-Santos JLM, Talamás-Rohana P. 2001. In vitro indomethacin administration upregulates interleukin-12 production and polarizes the immune response towards a Th1 type in susceptible BALB/c mice infected with *Leishmania mexicana*. Parasite Immunol. 23(11):599–606. 10.1046/j.1365-3024.2001.00421.x 11703811

[cit0070] Plamadeala C, Gosain SR, Hischen F, et al. 2019. Bio-inspired microneedle design for efficient drug/vaccine coating. Biomed Microdevices. 22(1):8. 10.1007/s10544-019-0456-z 31845066 PMC6915113

[cit0071] Ponte-Sucre A, Gamarro F, Dujardin J, et al. 2017. Drug resistance and treatment failure in leishmaniasis: a 21st century challenge. PLoS Negl Trop Dis. 11(12):e0006052. 10.1371/journal.pntd.0006052 29240765 PMC5730103

[cit0072] Pountain AW, Barrett MP. 2019. Untargeted metabolomics to understand the basis of phenotypic differences in amphotericin B-resistant *Leishmania parasites*. Wellcome Open Res. 4:176. 10.12688/wellcomeopenres.15452.1 32133420 PMC7041363

[cit0073] Pountain AW, Weidt SK, Regnault C, et al. 2019. Genomic instability at the locus of sterol C24-methyltransferase promotes amphotericin B resistance in *Leishmania parasites*. PLoS Negl Trop Dis. 13(2):e0007052. 10.1371/journal.pntd.0007052 30716073 PMC6375703

[cit0074] Prausnitz MR, Langer R. 2008. Transdermal drug delivery. NatBi. 26(11):1261–1268. 10.1038/nbt.1504 PMC2700785 18997767

[cit0075] Rodríguez Galvis MC, Pérez Franco JE, Casas Vargas MY, et al. 2020. Effectiveness and safety of amphotericin B deoxycholate, amphotericin B colloidal dispersion, and liposomal amphotericin B as third-line treatments for cutaneous and mucocutaneous leishmaniasis: a retrospective study. Am J Trop Med Hyg. 102(2):274–279. 10.4269/ajtmh.18-0514 31820708 PMC7008334

[cit0076] Rosas LE, Keiser T, Barbi J, et al. 2005. Genetic background influences immune responses and disease outcome of cutaneous L. Mexicana infection in mice. Int Immunol. 17(10):1347–1357. 10.1093/intimm/dxh313 16141242

[cit0077] Ryan E, Garland MJ, Singh TRR, et al. 2012. Microneedle-mediated transdermal bacteriophage delivery. Eur J Pharm Sci Off J Eur Feder Pharm Sci. 47(2):297–304. 10.1016/j.ejps.2012.06.012 PMC3778942 22750416

[cit0078] Sabri AH, Ogilvie J, Abdulhamid K, et al. 2019. Expanding the applications of microneedles in dermatology. Eur J Pharmaceut Biopharmaceut. 140:121–140. 10.1016/j.ejpb.2019.05.001 31059780

[cit0079] Samant PP, Niedzwiecki MM, Raviele N, et al. 2020. Sampling interstitial fluid from human skin using a microneedle patch. Sci Transl Med. 12(571). 10.1126/scitranslmed.aaw0285 PMC7871333 33239384

[cit0080] Sattler E, Kästle R, Welzel J. 2013. Optical coherence tomography in dermatology. JBO. 18(6):061224. 10.1117/1.JBO.18.6.061224 23314617

[cit0081] Schwing A, Pisani DF, Pomares C, et al. 2021. Identification of adipocytes as target cells for *Leishmania infantum* parasites. Sci Rep. 11. 10.1038/s41598-021-00443-y PMC8553825 34711872

[cit0082] Shrestha P, Stoeber B. 2018. Fluid absorption by skin tissue during intradermal injections through hollow microneedles. Sci Rep. 8(1), 10.1038/s41598-018-32026-9 PMC6137045 30213982

[cit0083] Shukla S, Huston RH, Cox BD, et al. 2022. Transdermal delivery via medical device technologies. Expert Opin Drug Deliv. 19(11):1505–1519. 10.1080/17425247.2022.2135503 36222232

[cit0084] Smith S, Witkowski A, Moghul A, et al. 2012. Compromised mitochondrial fatty acid synthesis in transgenic mice results in defective protein lipoylation and energy disequilibrium. PLoS One. 7(10):e47196. 10.1371/journal.pone.0047196 23077570 PMC3471957

[cit0085] Virginia Tech. 2017. SOP: Blood Collection in the Mouse, Tail Vein. https://ouv.vt.edu/content/dam/ouv_vt_edu/sops/small-animal-biomedical/sop-mouse-blood-collection-tail-vein.pdf

[cit0086] Sousa LMA, Carneiro MBH, Resende ME, et al. 2014. Neutrophils have a protective role during early stages of *Leishmania amazonensis* infection in BALB/c mice. Parasite Immunol. 36(1):13–31. 10.1111/pim.12078 24102495 PMC4307027

[cit0087] Sousa-Batista AJ, Pacienza-Lima W, Arruda-Costa N et al. 2018a. Depot subcutaneous injection with chalcone CH8-Loaded Poly(Lactic-Co-Glycolic Acid) microspheres as a single-dose treatment of cutaneous leishmaniasis. Antimicrob Agents Chemother. 62(3):e01822. 10.1128/AAC.01822-17 29263064 PMC5826136

[cit0088] Sousa-Batista AJ, Arruda-Costa N, Rossi-Bergmann B, et al. 2018b. Improved drug loading via spray drying of a chalcone implant for local treatment of cutaneous leishmaniasis. Drug Dev Ind Pharm. 44(9):1473–1480. 10.1080/03639045.2018.1461903 29618227

[cit0089] Sousa-Batista AJ, Pacienza-Lima W, Ré MI, et al. 2019. Novel and safe single-dose treatment of cutaneous leishmaniasis with implantable amphotericin B-loaded microparticles. Int J Parasitol Drugs Drug Resistance. 11:148–155. 10.1016/j.ijpddr.2019.06.001 PMC6904829 31331828

[cit0090] Sousa-Batista AJ, Arruda-Costa N, Escrivani DO, et al. 2020. Single-dose treatment for cutaneous leishmaniasis with an easily synthesized chalcone entrapped in polymeric microparticles. Parasitology. 147(9):1032–1037. 10.1017/S0031182020000712 32364107 PMC10317656

[cit0091] Staats J. 2019. Guidelines for gating flow cytometry data for immunological assays, in immunophenotyping: methods and protocols. In: McCoy J. J. P., editor. Springer: New York, NY. p 81. 10.1007/978-1-4939-9650-6_5 31522414

[cit0092] Tavares GSV, Mendonça DV, Lage DP, et al. 2019. In vitro and in vivo antileishmanial activity of a fluoroquinoline derivate against *Leishmania infantum* and *Leishmania amazonensis* species. Acta Trop. 191:29–37. 10.1016/j.actatropica.2018.12.036 30586571

[cit0093] Thomas BN, Buxbaum LU. 2008. FcgammaRIII mediates immunoglobulin G-induced interleukin-10 and is required for chronic *Leishmania mexicana* lesions. Infect Immun. 76(2):623–631. 10.1128/IAI.00316-07 18070890 PMC2223473

[cit0094] Torrentera FA, Aguilar Torrentera F, Lambot M, et al. 2002. Parasitic load and histopathology of cutaneous lesions, lymph node, spleen, and liver from BALB/c and C57BL/6 mice infected with *Leishmania mexicana*. Am J Trop Med Hyg. 66(3):273–279. 10.4269/ajtmh.2002.66.273 12139220

[cit0095] Untergasser A, Ruijter JM, Benes V, et al. 2021. Web-based LinRegPCR: application for the visualization and analysis of (RT)-qPCR amplification and melting data. BMC Bioinform. 22(1):398. 10.1186/s12859-021-04306-1 PMC8386043 34433408

[cit0096] Valigurová A, Kolářová I. 2023. Unrevealing the mystery of latent leishmaniasis: what cells can host leishmania? Pathogens. 12(2):246. 10.3390/pathogens12020246 36839518 PMC9967396

[cit0097] van der Maaden K, Trietsch SJ, Kraan H et al. 2014. Novel hollow microneedle technology for depth-controlled microinjection-mediated dermal vaccination: a study with polio vaccine in rats. Pharm Res. 31(7):1846–1854. 10.1007/s11095-013-1288-9 24469907

[cit0098] Varikuti S, Oghumu S, Saljoughian N, et al. 2017. Topical treatment with nanoliposomal amphotericin B reduces early lesion growth but fails to induce cure in an experimental model of cutaneous leishmaniasis caused by *Leishmania mexicana*. Acta Trop. 173:102–108. 10.1016/j.actatropica.2017.06.004 28602835 PMC5731240

[cit0099] Volpedo G, Huston RH, Holcomb EA, et al. 2021. From infection to vaccination: reviewing the global burden, history of vaccine development, and recurring challenges in global leishmaniasis protection. Expert Rev Vaccines. 20(11):1431–1446. 10.1080/14760584.2021.1969231 34511000

[cit0100] Volpedo G, Pacheco-Fernandez T, Holcomb EA, et al. 2021. Mechanisms of immunopathogenesis in cutaneous leishmaniasis and post kala-azar dermal leishmaniasis (PKDL). Front Cell Infect Microbiol. 11:685296. 10.3389/fcimb.2021.685296 34169006 PMC8217655

[cit0101] Volpedo G, Pacheco-Fernandez T, Holcomb EA, et al. 2022. Centrin-deficient *Leishmania mexicana* confers protection against new world cutaneous leishmaniasis. NPJ Vaccines. 7(1):32. 10.1038/s41541-022-00449-1 35236861 PMC8891280

[cit0102] Vos T, Lim SS, Abbafati C, et al. 2020. Global burden of 369 diseases and injuries in 204 countries and territories, 1990–2019: a systematic analysis for the global burden of disease study 2019. Lancet. 396(10258):1204–1222. 10.1016/S0140-6736(20)30925-9 33069326 PMC7567026

[cit0103] de Vries HJC, Schallig HD. 2022. Cutaneous leishmaniasis: a 2022 updated narrative review into diagnosis and management developments. Am J Clin Dermatol. 23(6):823–840. 10.1007/s40257-022-00726-8 36103050 PMC9472198

[cit0104] Waki K, Dutta S, Ray D, et al. 2007. Transmembrane molecules for phylogenetic analyses of pathogenic protists: leishmania-specific informative sites in hydrophilic loops of Trans- endoplasmic reticulum N-Acetylglucosamine-1-Phosphate transferase. Eukaryot Cell. 6(2):198–210. 10.1128/EC.00282-06 17142569 PMC1797956

[cit0105] Wang J, Li Y, Reguera RM, et al. 2025. Lymphatic-targeted amphotericin B nanocrystals delivered using microarray patches applied to cutaneous leishmaniasis. J Control Release. 386:114115. 10.1016/j.jconrel.2025.114115 40796014

[cit0106] Wasan E, Mandava T, Crespo-Moran P, et al. 2022. Review of novel oral amphotericin B formulations for the treatment of parasitic infections. Pharmaceutics. 14(11):2316. 10.3390/pharmaceutics14112316 36365135 PMC9697626

[cit0107] Weiss DJ, Nelson A, Vargas-Ruiz CA, et al. 2020. Global maps of travel time to healthcare facilities. Nat Med. 26(12):1835–1838. 10.1038/s41591-020-1059-1 32989313

[cit0108] Wermeling DP, Banks SL, Hudson DA, et al. 2008. Microneedles permit transdermal delivery of a skin-impermeant medication to humans. Proc Nat Acad Sci USA. 105(6):2058–2063. 10.1073/pnas.0710355105 18250310 PMC2538880

[cit0109] WHO | World Health Organization. 2022a. Global Health Observatory - Leishmaniasis. 2023/11/20/21:21:48; Available from: https://apps.who.int/neglected_diseases/ntddata/leishmaniasis/leishmaniasis.html

[cit0110] WHO. 2022b. Leishmaniasis. Available from: https://www.who.int/news-room/fact-sheets/detail/leishmaniasis

[cit0111] Wijnant G-J, Van Bocxlaer K, Yardley V, et al. 2018. Relation between skin pharmacokinetics and efficacy in AmBisome treatment of murine cutaneous leishmaniasis. Antimicrob Agents Chemother. 62(3):e02009. 10.1128/AAC.02009-17 29263075 PMC5826151

[cit0112] Yardley V, Croft SL. 2000. A comparison of the activities of three amphotericin B lipid formulations against experimental visceral and cutaneous leishmaniasis. Int J Antimicro Ag. 13(4):243–248. 10.1016/S0924-8579(99)00133-8 10755238

[cit0113] Zare MR, Khorram M, Barzegar S, et al. 2021. Dissolvable carboxymethyl cellulose/polyvinylpyrrolidone microneedle arrays for transdermal delivery of amphotericin B to treat cutaneous leishmaniasis. Int J Biiol Macromol. 182:1310–1321. 10.1016/j.ijbiomac.2021.05.075 34000308

[cit0114] Zhang Y, Chandra V, Riquelme Sanchez E, et al. 2020. Interleukin-17-induced neutrophil extracellular traps mediate resistance to checkpoint blockade in pancreatic cancer. J Exp Med. 217(12). 10.1084/jem.20190354 PMC7953739 32860704

