# Supplementary material for: Microneedle-based injection of Fungizone/Amphotericin B: an effective treatment for American cutaneous leishmaniasis in mice
Source: Drug Deliv. 2026 May 4;33(1):2665882. doi: 10.1080/10717544.2026.2665882 (PMC13148095; doi:10.1080/10717544.2026.2665882)
Supplement: Supplementary Material — Supplemental_Online_Material.docx [file IDRD_A_2665882_SM8164.docx]

**Supplemental Online Material:**


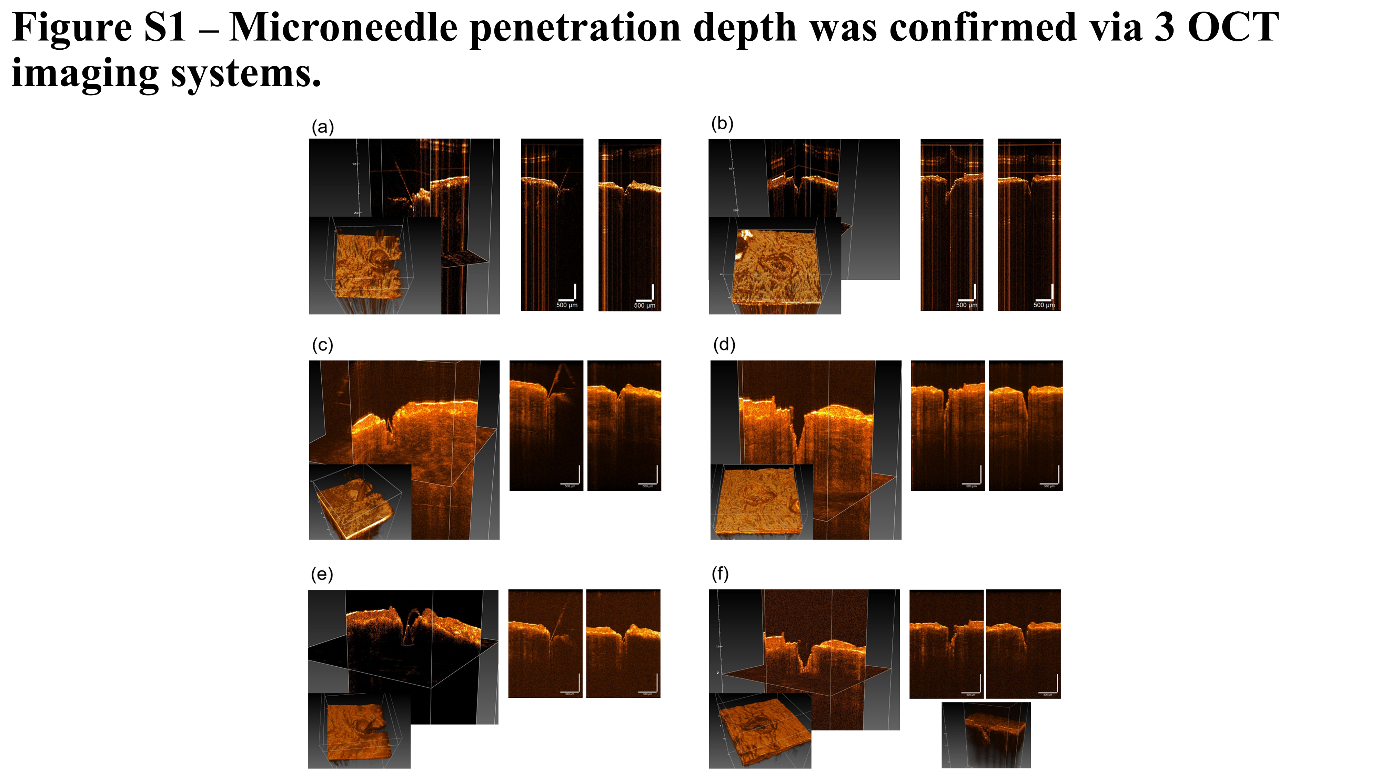


**Figure S1 – Microneedle penetration depth was confirmed via 3 OCT imaging systems**. Optical coherence tomography (OCT) cross-sectional images of microneedles in commercially-obtained porcine skin that were acquired using three Thorlabs OCT systems. (A) VEG210 system (1300 nm, 14 µm axial resolution) with microneedles inserted in skin. (B) VEG210 system showing skin after microneedle removal, with the puncture channel evident in the tissue. (C) TEL221 system (1300 nm, 5.5 µm axial resolution) with microneedles inserted in skin, showing improved resolution compared to the VEG210 system. (D) TEL221 system showing skin after microneedle removal, with the puncture channel clearly evident in the tissue. (E) GAN611 system (900 nm, 5.5 µm axial resolution, 8 µm lateral resolution) with microneedles inserted in skin, providing the highest lateral resolution among the three systems. (F) GAN611 system showing skin after microneedle removal, with the puncture channel clearly evident in the tissue. All OCT-LK3 series objectives were used with a 10 mm × 10 mm field of view. Scale bars: 500 µm.


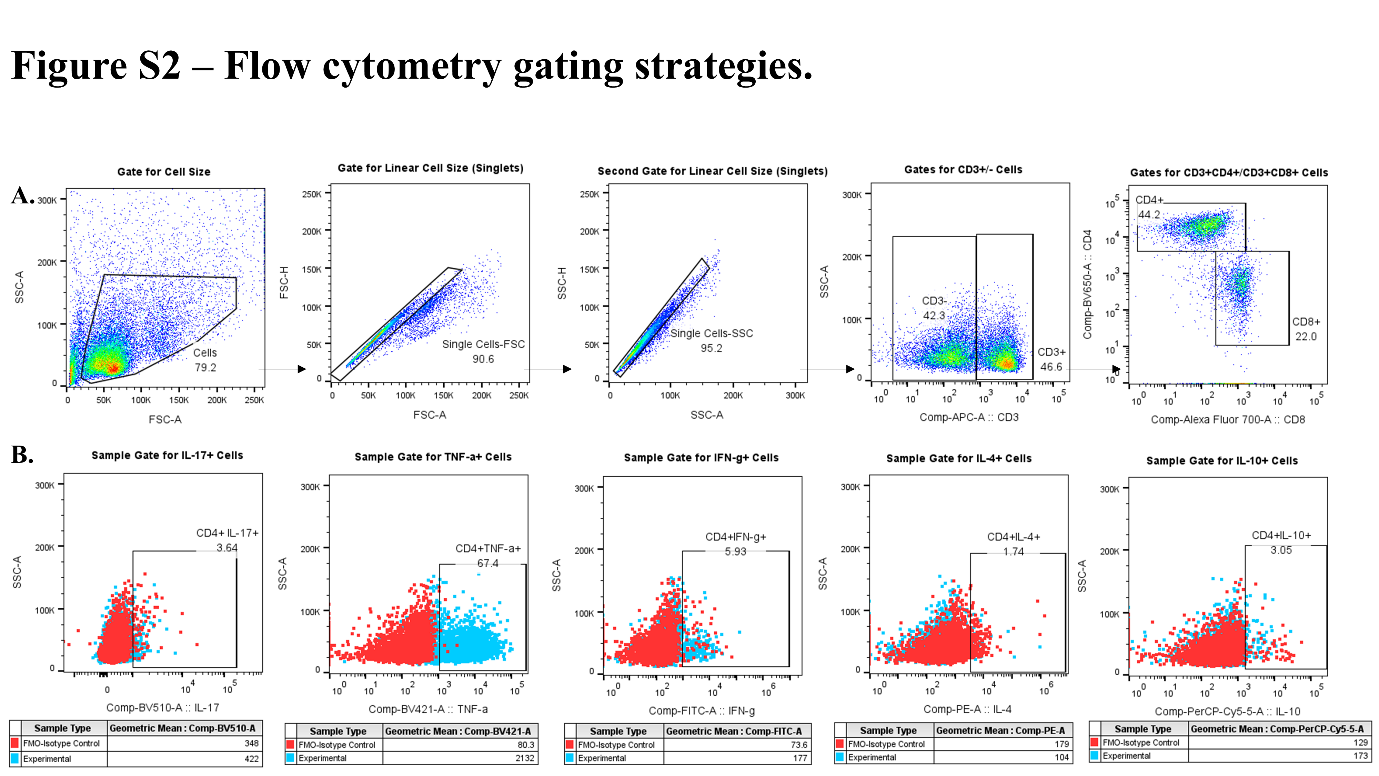


**Figure S2 – Flow Cytometry Gating Strategies.** All samples are from the 10-day microneedle trial. (A) Sample gates for cell size, linear characteristic in FSC and SSC directions (single cells), and common cell markers for CD3 (T-lymphocytes), and CD4/CD8 (T helper cells and cytotoxic T cells, respectively). (B) Sample gates for cytokine expression showing CD3+CD4+ single cells. In each case, the gate was placed based on concordance between the unstained and fluorescence-minus-one (FMO) isotype controls to minimize false-positives, even on cytokines not highly expressed. This was not possible only for IL-4, where the FMO control sample had a greater mean fluorescence intensity and a higher percent of false-positive cells than the percent of positive cells in the experimental samples in this channel, regardless of gate placement; thus, this channel was not used in analyses.


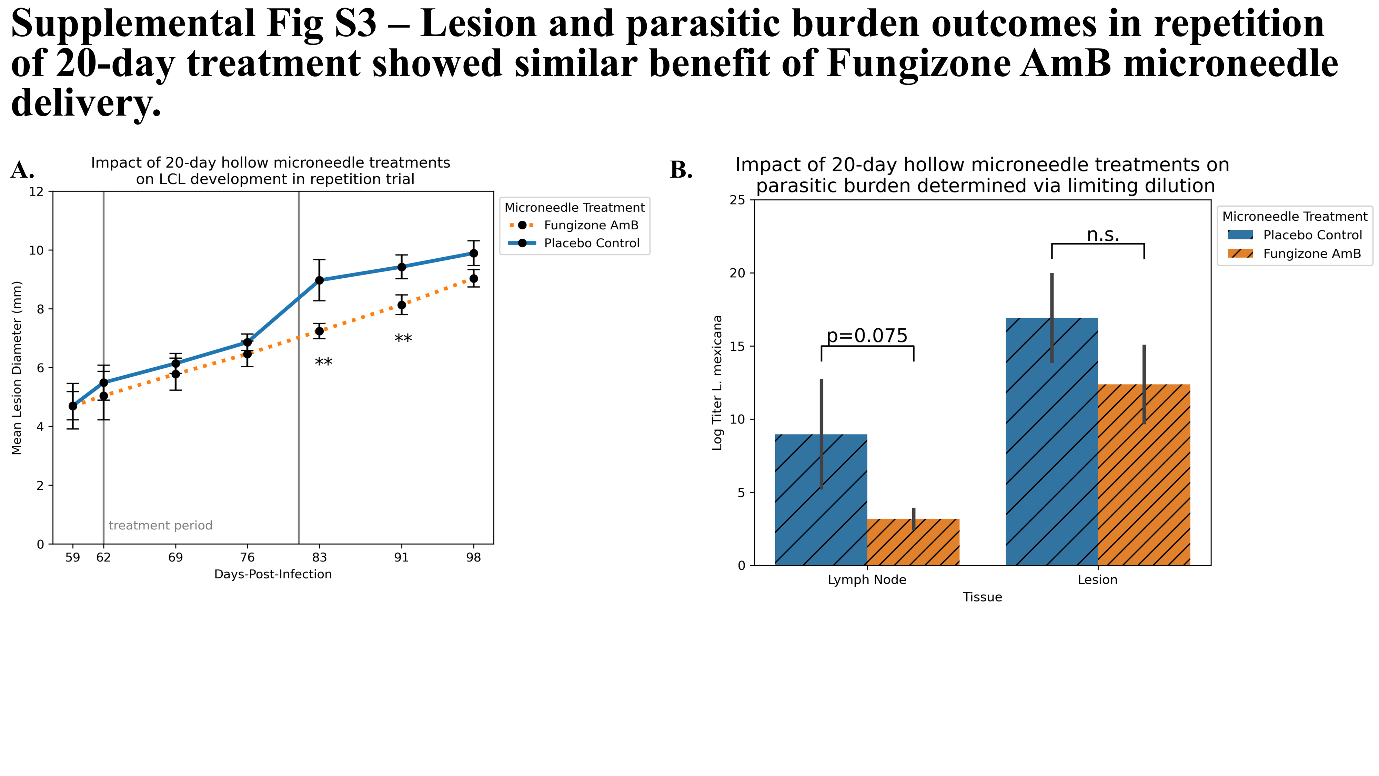


**Supplemental Fig S3 – Lesion and parasitic burden outcomes in repetition of 20-day treatment showed similar benefit of Fungizone AmB microneedle delivery.** (A) Lesion growth was again slowed in the Fungizone AmB-treated group over the placebo control group. After treatment cessation, a statistically significant difference in lesion size was observed for two consecutive weeks (p=0.028 for both, AUC=0.849-0.863). (B) Limiting dilution parasitic burden was employed in the repetition of the hollow microneedle 20-day treatment trial. At the time of euthanasia, lymph node tissue from the placebo control group trended towards elevated an *L. mexicana* log titer over the Fungizone AmB group (difference of log titer 5.8 or nearly 10^6^-fold, p=0.075, AUC=0.757). Lesion tissue also showed on average, a reduction in the drug-treated group of log titer 4.5 (10^4.5^–fold), but the difference was not significant (p=0.377, AUC=0.697).


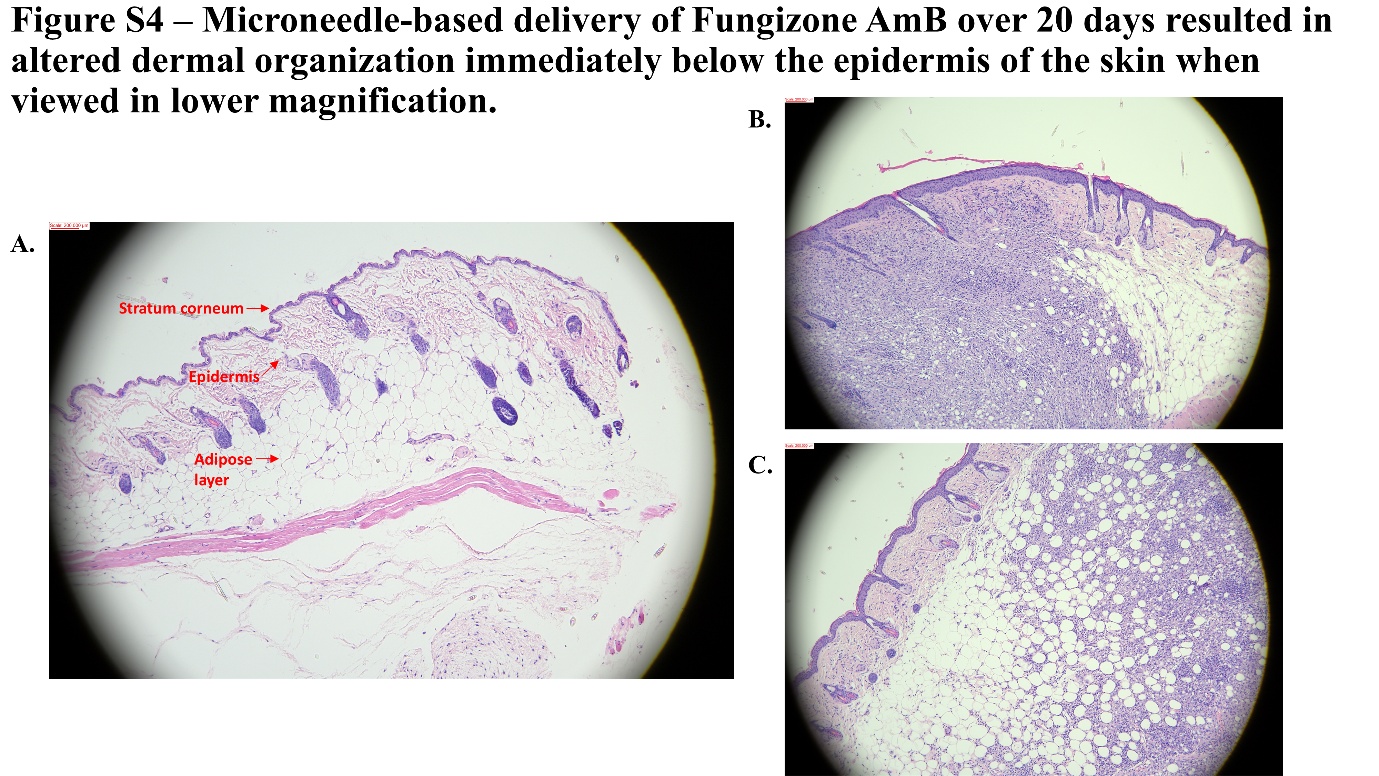


**Figure S4 – Microneedle-based delivery of Fungizone AmB over 20 days resulted in altered dermal organization immediately below the epidermis of the skin when viewed in lower magnification.** These 100X histology images are from the skin lesion tissue of the 20-day repetition microneedle trial. All show the superior stratum corneum skin to the deeper adipose tissue layers proximal to the epidermis and show an average view of the group’s appearance in this region of interest. This magnification highlights white blood cell infiltration and relative adipose tissue/vacuole organization. (A) Naïve (uninfected and no treatment) mouse, showing healthy skin layers. (B) Placebo-treated mouse, showing highly infected subepidermal zone right up to the dermis layer in some areas of the lesion. Smaller vacuole cells are apparent mainly within the edge of the lesion near the neighboring adipose tissue. High white blood cell infiltration is also seen throughout the lesion. (C) Fungizone AmB-treated mouse, showing lesser white blood cell presence in superficial layers of the skin. This finding is evident through near-normal epidermis and adipose layers above the deeper infected region. The observation of larger and more abundant vacuole cells in the lesions was heterogeneous throughout the lesion, but more predominant in the drug treatment group.
